# The KLP-7 Residue S546 Is a Putative Aurora Kinase Site Required for Microtubule Regulation at the Centrosome in *C*. *elegans*

**DOI:** 10.1371/journal.pone.0132593

**Published:** 2015-07-13

**Authors:** Xue Han, Kelly Adames, Ellen M. E. Sykes, Martin Srayko

**Affiliations:** Department of Biological Sciences, University of Alberta, Edmonton, Alberta, Canada; Institut de Génétique et Développement de Rennes, FRANCE

## Abstract

Regulation of microtubule dynamics is essential for many cellular processes, including proper assembly and function of the mitotic spindle. The kinesin-13 microtubule-depolymerizing enzymes provide one mechanism to regulate microtubule behaviour temporally and spatially. Vertebrate MCAK locates to chromatin, kinetochores, spindle poles, microtubule tips, and the cytoplasm, implying that the regulation of kinesin-13 activity and subcellular targeting is complex. Phosphorylation of kinesin-13 by Aurora kinase inhibits microtubule depolymerization activity and some Aurora phosphorylation sites on kinesin-13 are required for subcellular localization. Herein, we determine that a *C*. *elegans* deletion mutant *klp-7(tm2143)* causes meiotic and mitotic defects that are consistent with an increase in the amount of microtubules in the cytoplasmic and spindle regions of meiotic embryos, and an increase in microtubules emanating from centrosomes. We show that KLP-7 is phosphorylated by Aurora A and Aurora B kinases *in vitro*, and that the phosphorylation by Aurora A is stimulated by TPXL-1. Using a structure-function approach, we establish that one putative Aurora kinase site, S546, within the C-terminal part of the core domain is required for the function, but not subcellular localization, of KLP-7 *in vivo*. Furthermore, FRAP analysis reveals microtubule-dependent differences in the turnover of KLP-7(S546A) and KLP-7(S546E) mutant proteins at the centrosome, suggesting a possible mechanism for the regulation of KLP-7 by Aurora kinase.

## Introduction

The microtubule (MT) cytoskeleton is required for multiple essential cellular processes including cell division, polarization, and transportation of intracellular cargo. Remodeling of MT structures occurs through changes in MT growth and shrinkage, via the addition or removal of α/β tubulin subunits. The regulation of MT dynamics occurs spatially and temporally as demands on the MT cytoskeleton change throughout the cell cycle. Many proteins interact with MTs to regulate polymer behaviour, for example, the microtubule-associated protein XMAP215 promotes MT polymerization [1–3] whereas MCAK (mitotic centromere-associated kinesin) induces MT depolymerization [4–6]. A complete understanding of the MT-regulators is key to understanding how cells assemble and disassemble complex MT structures.

Mammalian MCAK and *Xenopus* XKCM1 are members of the MT-depolymerizing kinesin-13 family. Unlike conventional kinesins that move along MTs, kinesin-13 proteins utilize energy from ATP hydrolysis to depolymerise MTs [4, 7]. During mitosis, MCAK locates to many diverse regions of the cell, including the centromeres, kinetochores and spindle poles [8–13]. Subcellular regulation of MCAK activity in the cell influences the formation and persistence of the MTs at specific locations during spindle assembly. In vertebrates, MCAK is required to correct errors in spindle assembly that involve inappropriate kinetochore-MT attachments. Upon activation of MCAK, the mis-attached MTs are depolymerized so that new MT attachments can occur [6, 11, 14, 15], [8, 10, 16, 17].

The precise spatial regulation of human and *Xenopus* kinesin-13 proteins occurs in part through phosphorylation by Aurora A and Aurora B serine/threonine kinases. Aurora A kinase locates to the centrosomes and is involved in centrosome maturation and spindle assembly [18–21]. In contrast, Aurora B kinase locates to the centromere to regulate chromosome segregation and cytokinesis [22]. A subcellular division of labour for the Aurora kinases would allow independent regulation of substrates that locate to both chromatin and centrosomes, such as the kinesin-13 MT-depolymerases. Indeed, current data suggest that vertebrate kinesin-13s are negatively regulated by Aurora kinases at both chromatin and spindle poles [8, 10, 16, 17, 23].

In *C*. *elegans*, the kinesin-13 member, KLP-7 has been implicated in various MT-dependent processes. *klp-7(RNAi)* embryos exhibit a dramatic “spindle-break” phenotype in the first mitosis, whereby anaphase initiates abruptly and chromatids segregate rapidly. *klp-7(RNAi)* embryos appear to have fewer spindle midzone MTs in mitosis [24] and approximately 2-fold more astral MTs [25, 26], indicating a role for KLP-7 in regulating MTs. KLP-7 is detected at the holocentric kinetochores and also at the centrosomes in mitotic embryos [25, 27, 28], and KLP-7’s chromosomal location requires the kinetochore components CENP-A and CENP-C [27]. It is still unclear how KLP-7 is regulated at the kinetochore and the centrosome to facilitate proper mitotic spindle assembly and function.

Although Aurora kinases clearly influence kinesin-13 activity/location in other systems, this has not been established for KLP-7. The *C*. *elegans* genome encodes both Aurora A kinase (AIR-1) and Aurora B kinase (AIR-2) [29, 30]. Depletion of the Aurora kinases results in complex phenotypes, thus it has been difficult to determine their role in regulating KLP-7 via genetic epistasis experiments.

In this study, we show that a deletion mutant of *C*. *elegans klp-7* results in ectopic MT growth, affecting both meiotic and mitotic spindle function. Using a combination of *in vivo* and *in vitro* assays, we verify that KLP-7 is a substrate of Aurora kinase. Through mutational analysis, we identified one conserved Aurora phosphorylation site (S546) that is essential for KLP-7 function *in vivo*, but not for intracellular location of the protein. Furthermore, using FRAP, we determined that the phosphorylation state of this residue influences the dynamic behaviour of the KLP-7 protein at the centrosomes. Our data provide new insights on how KLP-7 could be regulated by Aurora kinases *in vivo*.

## Results

### Loss of KLP-7 results in increased microtubule polymer levels and spindle defects in meiosis and mitosis

**Fig 1:**
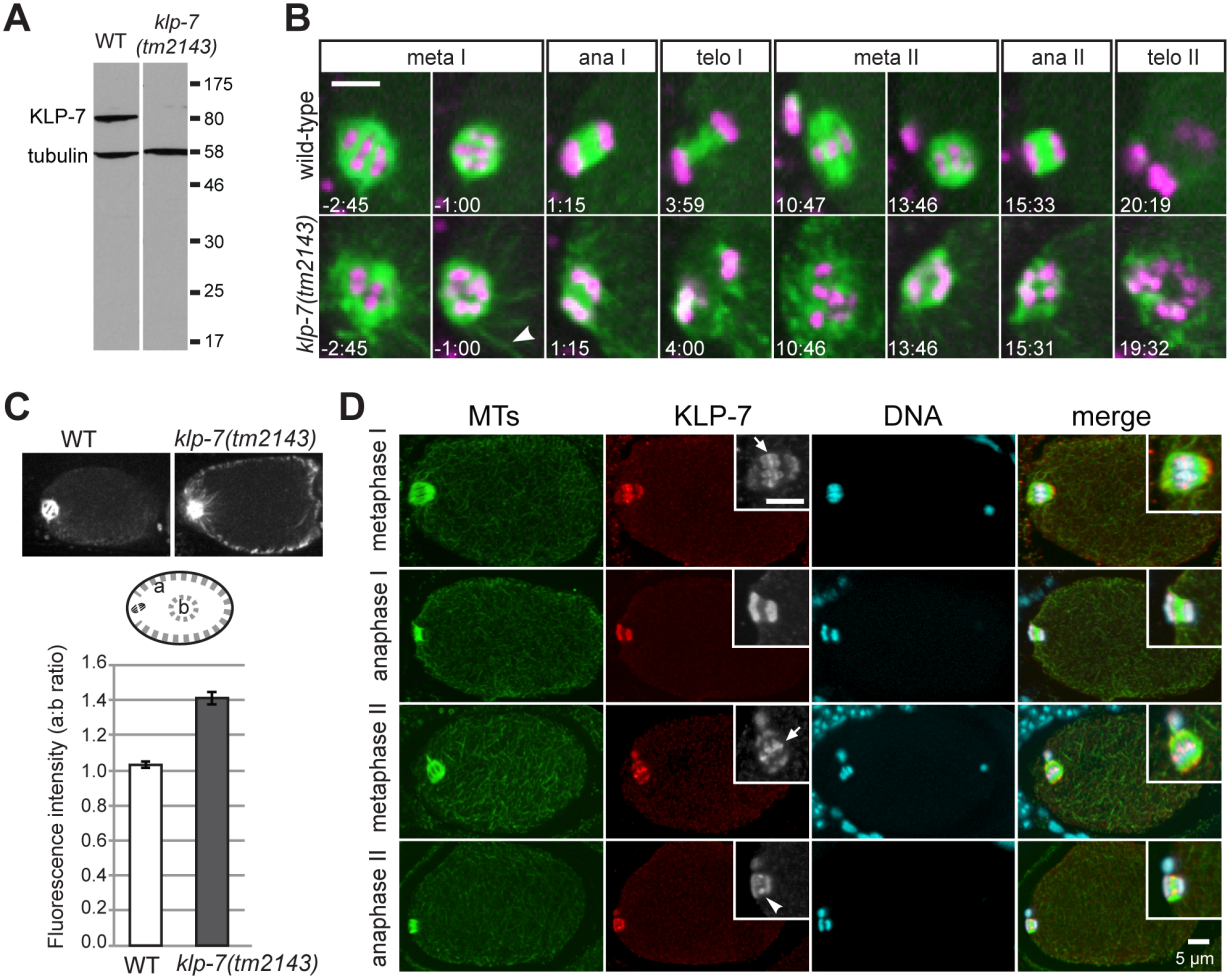
KLP-7 locates to female meiotic spindles and limits MT growth near the spindle and cortex. (A) A western blot probed for tubulin and KLP-7 demonstrates that KLP-7 is not detected in *klp-7(tm2143)* worm lysate. (B) Wild-type (upper panels, also S1 Movie) and *klp-7(tm2143)* (lower panels, also S2 Movie) meiotic spindles during meiosis I and II are displayed. GFP::tubulin is green and mCherry::histone is magenta. In *klp-7(tm2143)* embryos, many long MTs were visible near the meiotic spindle (arrowhead), and chromosome scattering was often observed after anaphase. Scale bar is 5 μm. (C) MTs were more prevalent near the cortex in meiotic *klp-7(tm2143)* embryos. The average ratio of cortical GFP::tubulin to a central region (a:b) in metaphase embryos is shown for WT (n = 16) and *klp-7(tm2143)* (n = 24). Error bars are SEM. (D) Wild-type embryos were fixed and stained to visualize MTs (green), KLP-7 (red) and DNA (blue). KLP-7 associates with chromosomes and spindle poles. Higher magnification insets show a higher concentration of KLP-7 at the region between meiotic chromosomes in metaphase I and II (arrows), and to the spindle midzone between the separating chromosomes in anaphase II (arrowhead). Scale bars are 5 μm.

To characterize *klp-7* loss-of-function phenotypes, we examined the MT behaviour in *klp-7(tm2143)* embryos after fertilization through to the first mitosis. *tm2143* contains an 875 bp deletion spanning two exons that is expected to remove the first 48 amino acids of the kinesin motor domain [31] and introduce a frame-shift [32]. Therefore, even if the *klp-7(tm2143)* transcript were translated, it would not likely yield a functional KLP-7 protein. Anti-KLP-7 antibodies have been generated against an N-terminal region of the protein (a.a. 14–190 of K11D9.1a [27]); *tm2143* is expected to remove only the last 94 amino acids of this region. With these antibodies, KLP-7 was not detected in *klp-7(tm2143)* worms by western blot (Fig 1A) or immunofluorescence (S1 Fig), consistent with the idea that *tm2143* is a null mutation. Surprisingly, many progeny from *tm2143* homozygous worms survive to adulthood and are fertile. Furthermore, *tm2143* lethality was temperature-sensitive, with 35% embryonic viability at permissive temperatures and 8% at the restrictive temperature (Table 1). *klp-7(tm2143)* worms also exhibited a higher number of spontaneous male progeny (1% vs. 0.2% at 25°C), likely indicating meiotic defects due to nondisjunction of the X chromosome [33].

**Table 1:** Embryonic viability of *klp-7(tm2143)* at different temperatures.

Temperature (°C)	Viability (%)	SEM (%)	# of embryos
15	32.6	5.1	385
20	34.6	4.1	849
25	8.3	1.3	428

To observe the MT cytoskeleton and chromatin during female meiosis, we examined wild-type and *klp-7(tm2143)* embryos expressing GFP::tubulin and mCherry::histone transgenes by filming one-cell embryos *in utero*. Compared to wild-type, *klp-7(tm2143)* embryos exhibited ectopic MTs in regions surrounding the meiotic spindle (Fig 1B; S1 and S2 Movies), and near the cortex of the cell. To quantify the effect, we measured the amount of GFP-tubulin fluorescence near the cortex and compared it to a region in the center of the cell (Fig 1C). The ectopic MTs in *klp-7(tm2143)* embryos were observed throughout meiosis I and II. During wild-type anaphase I and II, a MT bundle normally forms between the segregating chromosomes and is restricted to the midzone region [34, 35]. In both anaphase I and II in *klp-7(tm2143)* embryos, many MTs were observed beyond the spindle midzone region. Although the chromosomes separated during both meiosis I and II (n = 18), the spatial distribution of chromosomes was more variable in *klp-7(tm2143)*. This was especially evident between telophase I and metaphase II, and during telophase II (Fig 1B). Polar body extrusion failed in many *klp-7(tm2143)* embryos (7/18 MI; 14/18 MII) compared to wild-type controls (0/11 MI; 1/11 MII).

We examined the subcellular location of KLP-7 in female meiosis by immunostaining. During meiosis I and II, KLP-7 localized to chromosomes, and appeared concentrated between paired chromosomes, similar to what has been observed for members of the chromosomal passenger complex [35]. We also observed KLP-7 concentrated at the spindle poles in metaphase I and II. KLP-7 remained associated with chromosomes in anaphase and telophase. During late anaphase II, KLP-7 also localized to the spindle midzone between separating chromosomes (Fig 1D). KLP-7 immunofluorescence was also visible throughout the cytoplasm of meiotic embryos, consistent with a role of KLP-7 in regulating cytoplasmic MTs during meiosis.

**Fig 2:**
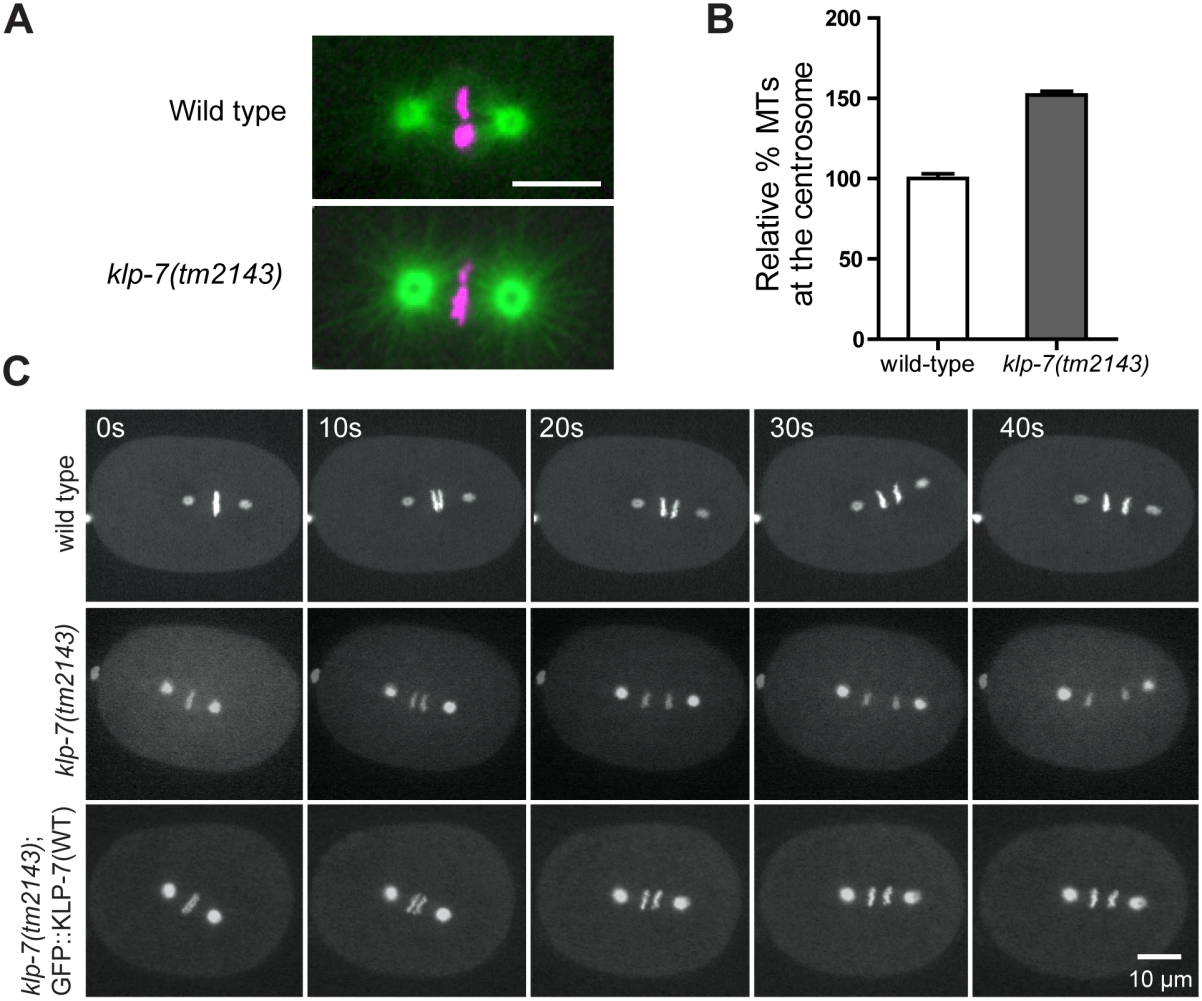
*klp-7(tm2143)* embryos exhibit increased levels of centrosomal MTs and a spindle-break phenotype during mitotic anaphase. (A) Representative images of wild-type and *klp-7(tm2143)* embryos expressing GFP::tubulin (green), mCherry::histone (magenta), in mitotic metaphase. Bar is 10 μm. (B) Quantification of centrosomal MTs in wild-type (n = 8) and *klp-7(tm2143)* (n = 12) embryos. The integrated intensity of GFP::tubulin per centrosome was measured. Levels are displayed relative to the average intensity of wild-type centrosomes (Error bars are SEM). (C) Still images of mitotic embryos from a time-lapse movie are shown. Top panel: wild-type embryo expressing GFP::γ-tubulin, GFP::histone (S3 Movie). Middle panel: *klp-7(tm2143)* embryo expressing GFP::γ-tubulin, mCherry::histone (S4 Movie). Lower panel: *klp-7(tm2143)* embryo expressing GFP::KLP-7(WT) (S5 Movie).

**Fig 3:**
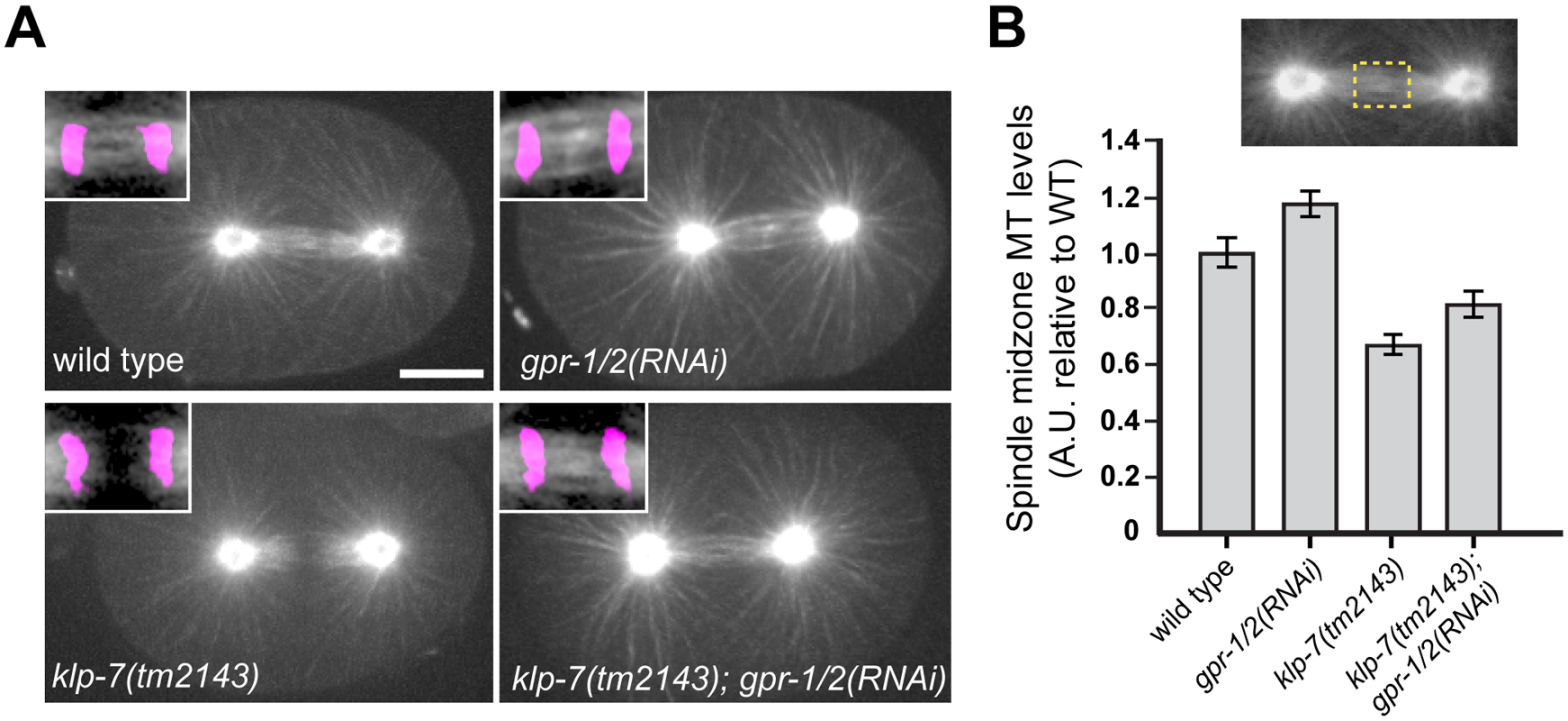
*gpr-1/2* RNAi increases spindle midzone MT levels in *klp-7(tm2143)* embryos. (A) Shown are representative images of wild-type, *gpr-1/2(RNAi)*, *klp-7(tm2143)* and *klp-7(tm2143); gpr-1/2(RNAi)* embryos at anaphase of mitosis. GFP::tubulin is white and mCherry::histone is magenta. (B) Quantification of GFP::tubulin fluorescence (arbitrary units, relative to WT) within a rectangular region at the midzone (inset) are shown for wild-type (n = 8), *gpr-1/2(RNAi)* (n = 9), *klp-7(tm2143)* (n = 12) and *klp-7(tm2143); gpr-1/2(RNAi)* (n = 12) embryos. Bar is 10 μm.

Previous examination of *klp-7(RNAi)* embryos in mitosis indicated three prevalent phenotypes: an increase in the number of astral MTs, an abrupt anaphase with higher than normal centrosome separation rates, and a reduction in the amount of spindle midzone MTs during anaphase [24–26]. We observed similar defects with the *klp-7(tm2143)* mutant (Figs 2 and 3; S3 and S4 Movies). *klp-7(tm2143)* mutant embryos that expressed a GFP::KLP-7(WT) transgene exhibited normal anaphase chromatid separation rates, indicating the utility of a structure-function approach to study KLP-7 *in vivo* (Fig 2; S5 Movie).

### KLP-7 is required for proper midzone microtubule formation

An increase in astral MT levels in *klp-7(tm2143)* embryos could cause increased pulling forces during anaphase, and thus explain the spindle breakage [26] (Fig 2A and 2B). However, these embryos also displayed fewer midzone MTs during anaphase (Fig 3A). Considering that KLP-7 locates to kinetochores, it is possible that KLP-7 contributes directly to the proper formation of the spindle midzone MTs [24, 28]. In this case, the spindle-break phenotype in *klp-7(tm2143)* embryos could be due to a weakened resistance to anaphase force. To distinguish between these possibilities, we reduced anaphase forces by depleting the G-protein regulator, GPR-1/2, in both wild-type and *klp-7(tm2143)* embryos. Consistent with previous studies [36–38], *klp-7(tm2143)* embryos showed a significant reduction in the amount of midzone MTs compared to wild type (Fig 3B, p = 0.0001; two-tailed Student’s t-test). RNAi against *gpr-1/2* in the *klp-7(tm2143)* background significantly increased midzone MTs compared with *klp-7(tm2143)* (p = 0.019; two-tailed Student’s t-test), and *gpr-1/2(RNAi)* embryos showed the highest level of midzone MTs (Fig 3B). It is well-established that *gpr-1/2(RNAi)* causes a reduction in anaphase pole separation rates [36–38], and our results further indicate that the amount of midzone MTs is inversely correlated with spindle-pulling force.

### KLP-7 is phosphorylated *in vivo*

In vertebrate systems, MCAK localization to chromatin and spindle poles is regulated via phosphorylation by Aurora B kinase and Aurora A kinase, respectively [8, 10, 16, 17, 23]. Aurora B kinase phosphorylates MCAK to inhibit MT depolymerization activity at the chromatin/kinetochore in cultured cells and *Xenopus* egg extracts [8, 10, 16] and Aurora A kinase phosphorylates MCAK at the spindle poles to promote Ran–dependent spindle bipolarity [23]. KLP-7 has twelve putative Aurora kinase sites, as determined by the group-based phosphorylation predicting and scoring method [39]. Therefore, we reasoned that the Aurora kinases could be important regulators of KLP-7 in *C*. *elegans*. In worms, Aurora B kinase (AIR-2) is a chromosomal passenger complex protein implicated in meiotic and mitotic spindle functions and Aurora A kinase (AIR-1) is involved in centrosome maturation and mitotic spindle assembly [29, 30, 40–44]. Because of the overlapping locations of KLP-7 with AIR-1 and AIR-2 at centrosomes and chromosomes respectively, we sought to determine whether Aurora kinases play a role in either localizing or regulating KLP-7. Severe phenotypes and pleiotropy associated with *air-1(RNAi)* and *air-2(RNAi)* prevented the use of a simple genetic experiment to determine if the Aurora kinases are in the same pathway as KLP-7 for MT regulation. Therefore, we used a structure-function analysis of KLP-7 to test the hypothesis that worm kinesin-13 is regulated by the Aurora kinases *in vivo*.

**Fig 4:**
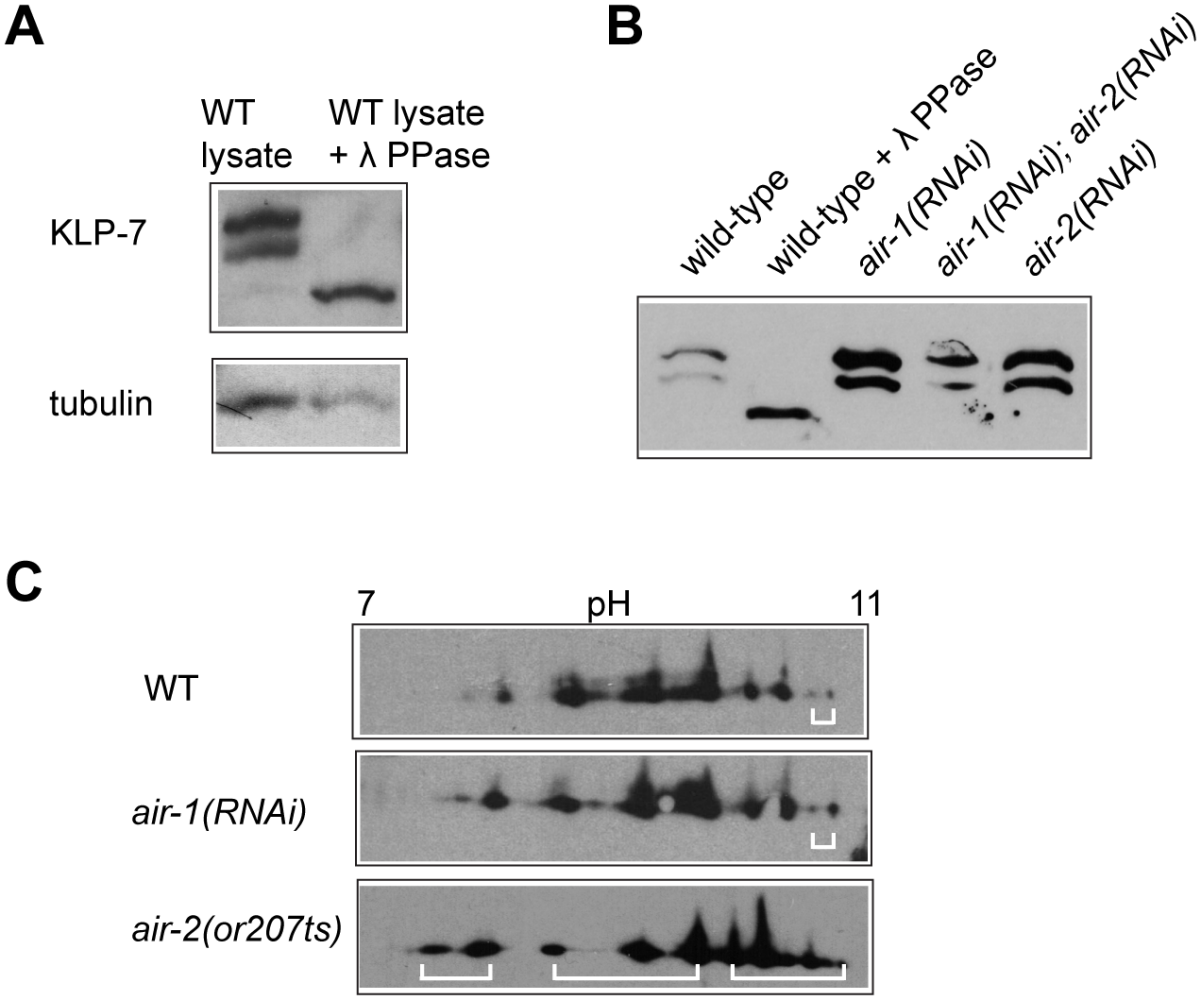
KLP-7 is phosphorylated *in vivo*. (A) A western blot analysis of wild-type and phosphatase-treated lysates separated by Phos-tag SDS PAGE, probed with anti-KLP-7 antibodies. (B) Phos-tag western blots of wild-type and kinase knockdown lysates, probed with anti-KLP-7. Twenty RNAi-treated young adult hermaphrodites were loaded into each lane. (C) 2D gel electrophoresis of KLP-7 in wild-type (top), *air-1(RNAi)* (middle) and *air-2(or207ts)* (bottom) lysates, followed by immunoblotting with anti-KLP-7 are shown. The pH gradient is indicated. Putative phospho-isoforms that display differences in wild-type and kinase knockdown lysates are shown with white brackets.

We first looked for evidence of *in vivo* phosphorylation of KLP-7 using Phos-tag SDS-PAGE followed by immunoblotting with anti-KLP-7 antibodies. Immunoblots of wild-type lysates revealed two bands that migrated more slowly than expected for KLP-7 (predicted MW of 80 KDa). Treatment of the lysate with protein phosphatase resulted in a single band at 80 KDa, which likely represented non-phosphorylated KLP-7 (Fig 4A). We concluded that the KLP-7 protein was phosphorylated *in vivo*, possibly at multiple sites.

To determine whether the mobility of KLP-7 was altered by *in vivo* Aurora kinase activity, we collected lysates from wild-type, *air-1(RNAi)*, *air-2(RNAi)* and *air-1(RNAi);air-2(RNAi)* worms. For each RNAi experiment, expected phenotypes were observed by fluorescence microscopy, confirming effective knockdown. Using this approach, we did not observe an obvious change in KLP-7 mobility on Phos-tag SDS PAGE (Fig 4B), suggesting that the RNAi knockdown did not significantly alter the phosphorylation state of KLP-7, or that subtle changes in phosphorylation occurred that were not detectable via the Phos-tag assay.

### Aurora kinase activity has a subtle effect on the mobility of endogenous KLP-7 via 2D gel electrophoresis

In order to resolve any differentially phosphorylated forms of KLP-7, we next looked for Aurora-dependent mobility shifts in KLP-7 via 2-D gel electrophoresis, followed by immunoblotting with anti-KLP-7 antibodies. Using this approach, we observed many distinct protein species (Fig 4C), which were sensitive to phosphatase treatment (S2 Fig). The migration pattern of KLP-7 suggested that endogenous KLP-7 protein might undergo multiple modifications. *air-1(RNAi)* resulted in a relative increase in at least one species of KLP-7, consistent with reduced phosphorylation (Fig 4C; right side of isoelectric axis). *air-2(or207ts)* caused many changes in the overall pattern of KLP-7 migration, some of which could represent increased or decreased phosphorylation. Although the complex migration pattern of KLP-7 suggested that other regulatory enzymes probably also modify the protein, we concluded that both Aurora kinases likely contribute to KLP-7 modification *in vivo*.

### AIR-1 and AIR-2 directly phosphorylate KLP-7 *in vitro*

**Fig 5:**
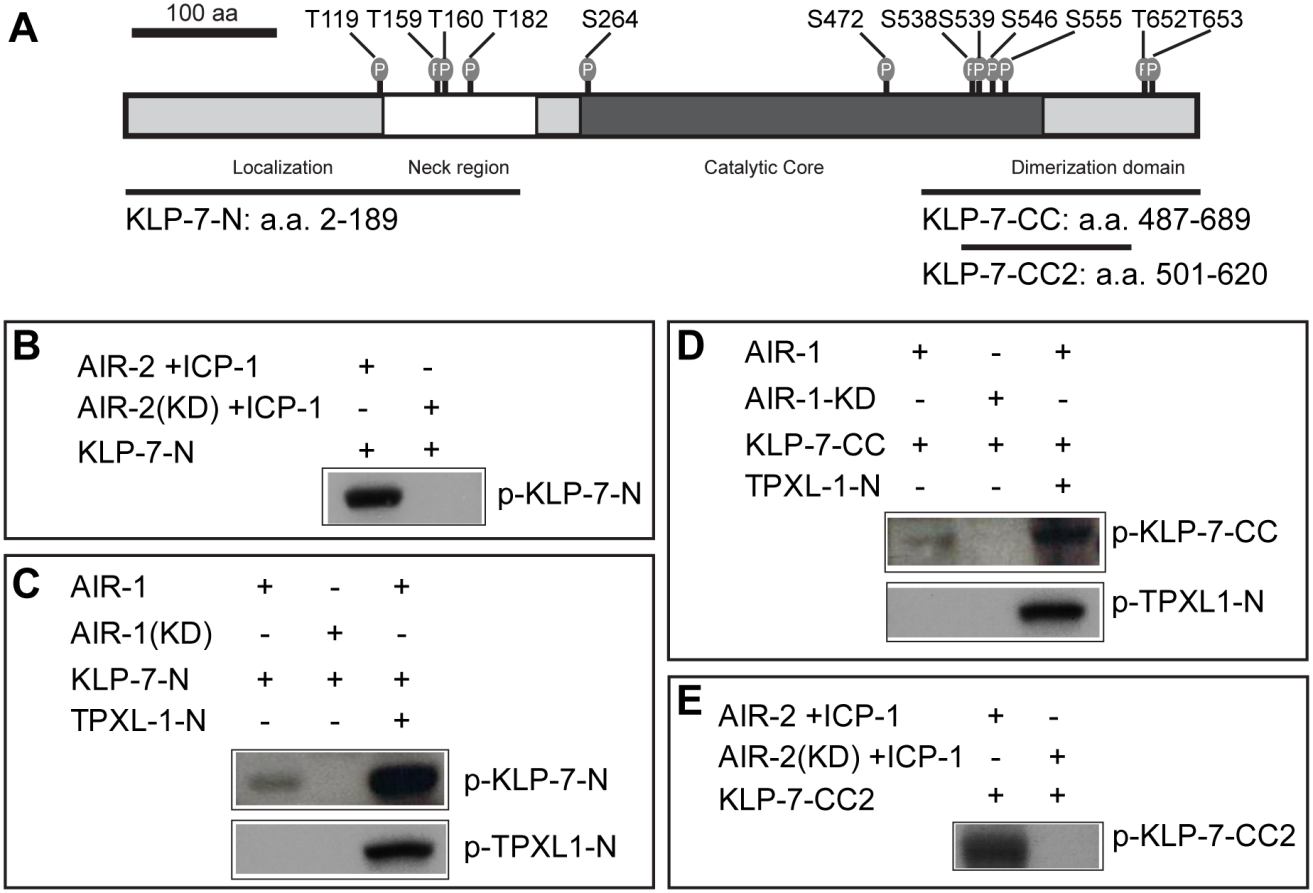
KLP-7 is phosphorylated by AIR-1 and AIR-2 kinases *in vitro*. (A) Shown is a schematic of the KLP-7 protein, with Aurora kinase phosphorylation sites (gray) predicted by GPS [39]. Fragments used for *in vitro* kinase reactions are indicated below. (B-E) AIR-1 or AIR-2 kinases were incubated with different substrates *in vitro* and γ[32P]-ATP incorporation was determined via SDS-PAGE and subsequent autoradiography. (B) AIR-2 kinase with coactivator ICP-1 phosphorylates KLP-7 within the N-terminus. (C-D) TPXL-1 N-terminal fragment (aa 1–63; [48]) stimulates AIR-1 phosphorylation of KLP-7 within the N-terminus and (D) the C-terminus. (E) AIR-2 kinase with coactivator ICP-1 phosphorylates KLP-7 within the C-terminus. KD = kinase dead; Ponceau-S stained membranes for B-E are provided in S3 Fig.

In order to verify that KLP-7 is an Aurora substrate, we performed *in vitro* kinase assays on bacterially-expressed GST-KLP-7 proteins. Full-length KLP-7 was insoluble, therefore, we expressed KLP-7 fragments that covered clusters of putative Aurora sites at the N-terminus KLP-7-N (a.a. 2–189) and the C-terminus with part of the core domain, KLP-7-CC (a.a. 487–689) (Fig 5A). We first tested the N-terminal fragment for phosphorylation. Previous work showed that the *C*. *elegans* INCENP protein, ICP-1, promoted AIR-2 phosphorylation of maltose binding protein [45]. Using an AIR-2/ICP-1 enzyme cocktail in an *in vitro* kinase assay, we observed that N-terminal KLP-7 was efficiently phosphorylated (Fig 5B and S3 Fig). In contrast, AIR-1 only weakly phosphorylated this fragment (Fig 5C and S3 Fig). This suggested that either KLP-7 was not an effective substrate for AIR-1 or that, similar to AIR-2 kinase, AIR-1 required an accessory protein for efficient *in vitro* activity. TPX2 (Targeting Protein for *Xenopus* Klp2) has been shown to target Aurora A to spindles in both human cells and *C*. *elegans*, and to promote Aurora A kinase activity on histone H3 *in vitro* [46–48]. AIR-1 and TPXL-1 co-localize along astral MTs, with enrichment near the centrosome [48]. We tested TPXL-1 for its ability to facilitate phosphorylation of KLP-7 by AIR-1 *in vitro*. Indeed, AIR-1 together with TPXL-1 efficiently phosphorylated the N-terminal KLP-7 fragment (Fig 5C and S3 Fig).

We repeated the kinase assays using a C-terminal fragment that contained part of the core domain KLP-7-CC (a.a. 487–689) and we observed similar TPXL-1-dependent activity for AIR-1 kinase (Fig 5D and S3 Fig), but phosphorylation by AIR-2 was not apparent (S4 Fig). Because the C-terminal fragment appeared less soluble in the bacterial lysate, and was less phosphorylated by AIR-1 compared to the N-terminal fragment, we concluded that this fragment might not be an effective substrate due to non-native conformation. Therefore, we tested a smaller fragment (CC2) and observed efficient phosphorylation by AIR-2 kinase (Fig 5E and S3 Fig). We concluded that both AIR-1 and AIR-2 directly phosphorylate KLP-7 *in vitro*, and that TPXL-1 promotes AIR-1-dependent phosphorylation of KLP-7.

### Mutational analysis of putative Aurora kinase sites reveals a role for S546 in KLP-7 function *in vivo*

In order to test the putative Aurora phosphorylation sites for their role in KLP-7 function *in vivo*, we mutated transgenes and tested their ability to rescue the *klp-7(tm2143)* deletion mutant. GFP-tagged KLP-7 was used to monitor changes in the intracellular location of the mutated proteins. We were unable to obtain every possible mutant combination, however, we tested multiple residues within clusters of kinase sites for both non-phosphorylation (serine or threonine to alanine, S/T to A) and constitutive phosphorylation (serine or threonine to glutamic acid, S/T to E) (S1 Table). We scored each of the stable transgenic lines for embryonic viability. At 25°C, *klp-7(tm2143)* worms exhibited low embryonic viability (15.9±4.1% survival). Compared to *klp-7(tm2143)*, transgenic worms, including KLP-7(WT), T119A, T119E, T182E, N4A, N4E, S539A, S539E, S538A539A, S538ES539E and 8A showed increased embryonic viability, ranging from 40% to 90% (Table 2). This suggested that these transgenes retained some function. In contrast, four transgenic worm strains exhibited very low embryonic viability (16–30%; Table 2). In all of these strains, the S546 residue was mutated (S546A, S546E, T182ES539ES546E, and 10E). Western blots of lysates from transgenic animals probed with KLP-7 revealed that the fusion proteins were expressed, suggesting that the loss of embryonic viability was not due to a lack of protein expression (S5 Fig).

**Table 2:** Embryonic viability of phospho-mutant KLP-7 transgenic worms at 25°C.

Genotype	Viability (%)	SEM (%)	p-value*	# of embryos
*klp-7 (tm2143)*	15.9	4.1	1	768
KLP-7 WT	84.6	6.9	0.0008	511
T119E	62.0	2.3	0.0003	508
T119A	87.7	3.7	6.20E-05	547
N4E	44.3	5.8	0.0092	331
N4A	76.0	3.2	0.0001	481
S538ES539E	49.5	6.9	0.0070	476
S538AS539A	48.6	3.8	0.0011	240
S539E	65.3	2.4	0.0002	677
S539A	84.1	3.1	1.18E-05	994
S546E	27.5	3.1	0.0530	1392
S546A	17.6	1.8	0.2662	464
T182ES539ES546E	15.7	2.7	0.9669	548
T182E	53.7	3.7	0.0005	597
8A	74.3	6.6	0.0005	361
10E	28.0	1.9	0.0670	861

*P-values associated with a Student's unpaired t-test, with a two-tailed distribution. Viability of each transgenic line was compared with that of *klp-7(tm2143)*.

**Fig 6:**
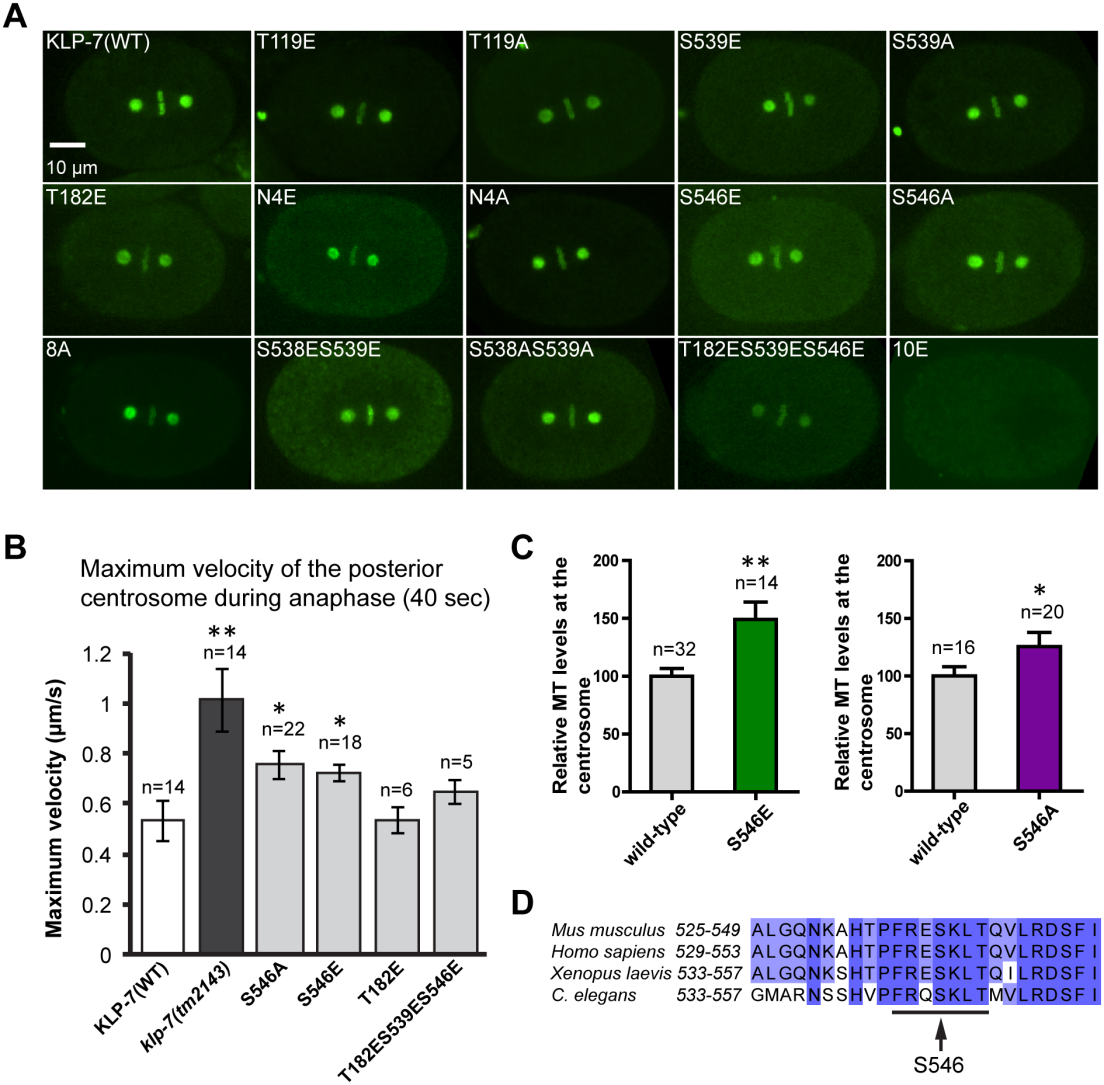
KLP-7 residue S546 is required for protein function but not subcellular localization. (A) KLP-7 proteins mutated at different predicted Aurora kinase phosphorylation sites localize at centrosomes and kinetochores. Images of metaphase embryos expressing GFP::KLP-7(WT) or GFP::KLP-7(phospho-mutant) transgenes are shown. (B) Different versions of KLP-7-GFP were expressed in *klp-7(tm2143)* worms. The maximum velocity of the posterior centrosome during the first 40 seconds of mitotic anaphase was determined; also see S7 Fig. The relevant alteration to the *klp-7* transgene is listed. Average maximum velocity was determined from GFP-KLP-7(WT), *klp-7(tm2143)*, GFP-KLP-7(S546A), GFP-KLP-7(S546E), GFP-KLP-7(T182E), and GFP-KLP-7(T182S539ES546E). *klp-7(tm2143)* control worms expressed GFP::γ-tubulin and GFP::histone to enable centrosome tracking; all other strains were tracked with the GFP::KLP-7 signal. GFP::KLP-7(S546E) and GFP::KLP-7(S546A) transgenes failed to rescue *klp-7(tm2143)*. P-values for WT vs experimental data were: *klp-7(tm2143)* P = 0.002; GFP-KLP-7(S546A) P = 0.017; GFP-KLP-7(S546E) P = 0.030; GFP-KLP-7(T182E) P = 0.498; GFP-KLP-7(T182S539ES546E) P = 0.135. ** (0.0005<P<0.005); * (0.005<p<0.05), Two-tailed Student’s t-test. (C) Similar to *klp-7(tm2143)*, GFP::KLP-7(S546E) and GFP::KLP-7(S546A) embryos exhibited an increase in centrosomal MTs, relative to WT. At least 14 centrosomes were analyzed for each strain. **(0.0005<P<0.005); *(0.005<p<0.05), Two-tailed Student’s t-test. (D) S546 is within a conserved Aurora kinase motif (underlined).

We next examined GFP::KLP-7 location in the various mutants using fluorescence microscopy (Fig 6A and S6 Fig). Our results indicated that S546A, S546E, and T182ES539ES546E fusion proteins exhibited intracellular localization patterns that were similar to wild-type GFP::KLP-7 (Fig 6A). In contrast, the GFP::KLP-7(10E) mutant, predicted to mimic constitutive phosphorylation at 10 putative Aurora kinase sites (S1 Table), exhibited only cytoplasmic fluorescence in the one-cell embryos.

The rescue experiments indicated that residue S546 was important for the function of KLP-7. Because KLP-7 activity inversely correlated with centrosomal MT levels and spindle pole separation rates in anaphase, we tested whether these parameters were specifically influenced by changes to S546. To quantify the effect, we measured the velocity of the posterior centrosome during the first 40 seconds of anaphase. We found that, while other mutant forms of KLP-7 rescued the fast centrosome movements that gave rise to the spindle-break phenotypes (S7 Fig), the GFP::KLP-7(S546A) and GFP::KLP-7(S546E) mutations still exhibited fast centrosome movements in anaphase (Fig 6B). Furthermore, neither mutation rescued the overproduction of centrosomal MTs characteristic of *klp-7(tm2143)* (Figs 2B and 6C). Therefore, the S546 residue is likely important for KLP-7 function at the centrosome.

**Fig 7:**
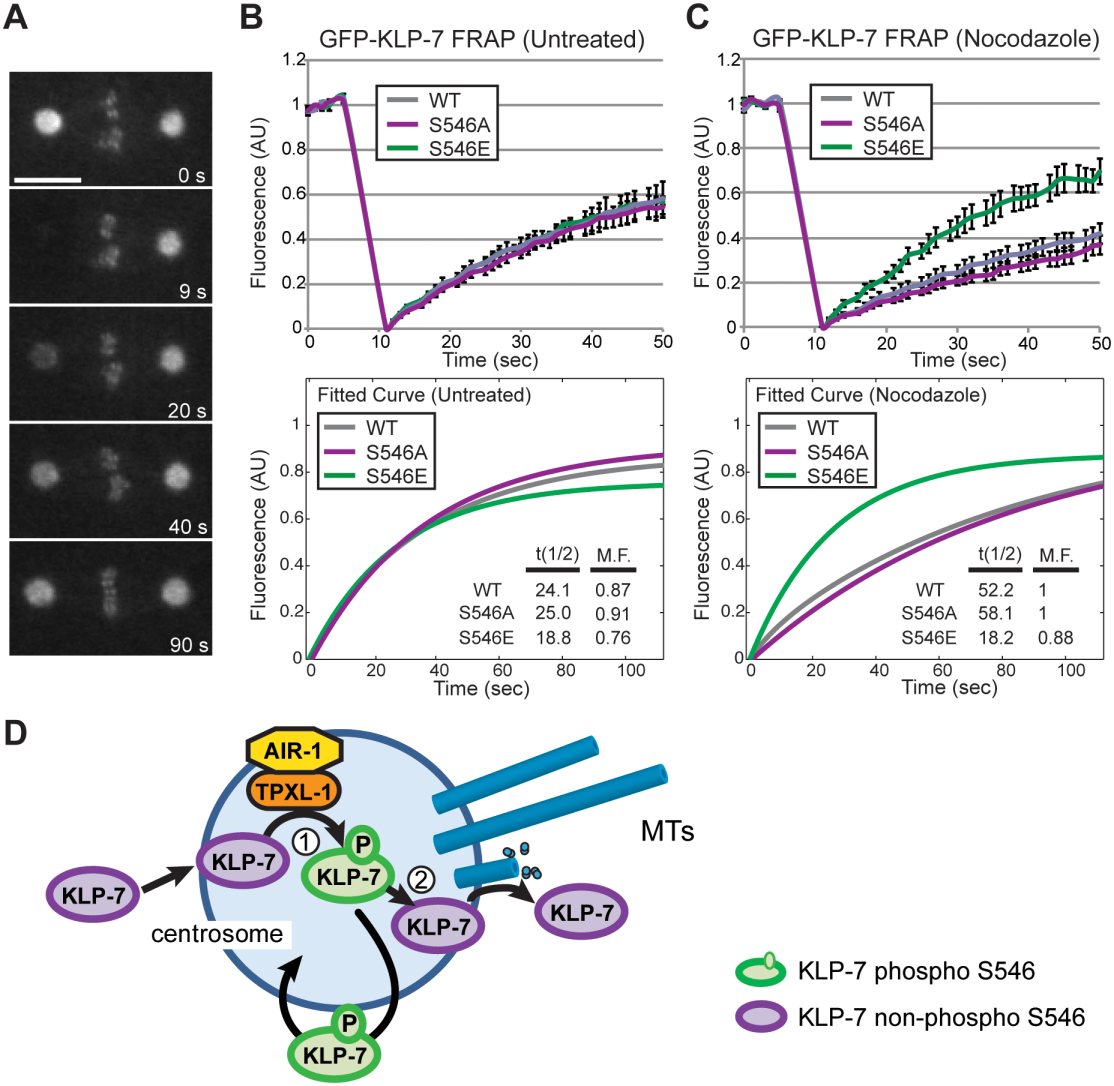
Fluorescence recovery after photobleaching of centrosomal GFP::KLP-7. (A) An example of fluorescence recovery of GFP::KLP-7 at the centrosome is shown. One centrosome (left) was photobleached and the fluorescence intensity was measured from images acquired at one second intervals. Bar: 10 μm. (B) Centrosomal recovery rates (in arbitrary units) are displayed for the first 50 sec for WT, S546A and S546E versions of GFP::KLP-7. Error bars are SEM (n ≥ 5). (C) Pre-treatment of the embryos with nocodazole revealed a slower recovery time for WT and S546A, compared to untreated embryos. S546E recovery is similar in nocodazole-treated and untreated samples. Normalized fitted recovery curves for B and C are shown below; also see S8 Fig. Insets display the calculated t(1/2) and mobile fractions (M.F.). (D) A model for two phases of KLP-7 behaviour related to the phosphorylation state of S546. Phosphorylated KLP-7 (green) exchanges rapidly between centrosomes and cytoplasm, by a MT-independent mechanism. Dephosphorylated KLP-7 (magenta) locates to the centrosome but must undergo phosphorylation (1) and subsequent dephosphorylation (2) before depolymerizing MTs and exiting the centrosome. The phosphorylation of KLP-7 at the centrosome could occur via TPXL-1-activated Aurora kinase A (AIR-1), based on *in vitro* data and data from Ozlü et al., [48].

Although the S546 mutants did not alter the localization of GFP::KLP-7, S546 could be important for dynamic KLP-7 behaviour not revealed by conventional microscopy. Therefore, we used fluorescence recovery after photobleaching (FRAP) to measure the turnover of KLP-7 at centrosomes (Fig 7A). Using this approach, we found similar recovery rates for GFP::KLP-7(WT) and GFP::KLP-7(S546A) (T1/2 = 24.1 and 25.0, respectively) and a slightly faster recovery for GFP::KLP-7(S546E) (T1/2 = 18.8, Fig 7B and S8 Fig). Because KLP-7 is expected to interact with MTs as part of its depolymerizing function, we next tested KLP-7 protein recovery at the centrosomes in embryos pre-treated with the MT-depolymerizing drug nocodazole. In the nocodazole-treated embryos, GFP::KLP-7(WT) and GFP::KLP-7(S546A) exhibited similar, but much slower recovery rates (T1/2 = 52.2 and 58.1, respectively), suggesting that KLP-7 turnover was at least partially dependent on centrosomal MTs. Interestingly, GFP::KLP-7(S546E) embryos exhibited a recovery rate similar to untreated embryos (T1/2 = 18.2, Fig 7C and S8 Fig). The FRAP results suggested that, at the centrosome, the turnover of both WT and S546A forms of KLP-7 were dependent on MTs, and that phosphorylation of S546 interfered with KLP-7-MT interactions at the centrosome.

## Discussion

### KLP-7 is required for the proper formation and organization of microtubules

Although *klp-7(tm2143)* is an apparent null mutation, it causes temperature-sensitive lethality. Because MT growth in *C*. *elegans* is inherently temperature-sensitive [26], it is conceivable that the loss of a MT regulator could also cause variable phenotypic severity at different temperatures. Our characterization of *klp-7(tm2143)* mutants indicated that *C*. *elegans* kinesin-13 normally limits MT formation within the cytoplasm and spindles during meiosis as well as at the centrosomes in mitosis. One exception to this generalization is that loss of KLP-7 activity also caused a decrease in spindle midzone MTs during anaphase of mitosis. KLP-7 could be required to generate the spindle midzone MTs directly, however, this property seems inconsistent with the expected molecular role of kinesin-13 proteins to depolymerize MTs. Alternatively, the reduction of spindle midzone MTs associated with KLP-7 loss could be an indirect consequence of increased pulling forces during anaphase [24, 25].

It has been previously proposed that anaphase spindle movements in *C*. *elegans* one-cell embryos utilize a limited number of cortically–located force generators [49]. In this context, excess microtubules due to *klp-7(tm2143)* might not be expected to increase the anaphase pulling force. However, *klp-7(tm2143)* could simply increase the rate at which new MTs contact a limiting number of cortical force generators. Indeed, it has been suggested that altering MT-contact frequency with the cortex could modulate anaphase spindle movements if individual MT-cortex interactions occur via a brief touch-and-pull mechanism [50].

Inhibition of MCAK causes defects in chromosome alignment in meiotic *Xenopus* egg extracts and mouse oocytes, as well as chromosome segregation defects resulting from increased MT-kinetochore attachment errors [14, 16, 51]. Loss of KLP-7 resulted in excessive cortical MTs in meiotic embryos and a disorganized MT network associated with female meiotic spindles. It is not clear what relationship, if any, the excess cortical MTs have to the embryonic lethality associated with *klp-7(tm2143)*. However, excess MTs near the spindle, regardless of their point of origin, could physically displace chromosomes and contribute to chromosome scattering observed in meiosis I and II.

### Phosphorylation of KLP-7 by Aurora kinases

Our data showed that endogenous KLP-7 is phosphorylated both *in vitro* and *in vivo*. Although the alteration to KLP-7 mobility upon AIR-1 loss was subtle, it is possible that only a subset of KLP-7 at the centrosomes is directly phosphorylated by AIR-1 *in vivo*. Alternatively, perhaps only one or a few potential KLP-7 sites are utilized by AIR-1 to regulate the protein, in which case only subtle mobility shifts would be expected via 2D gel analysis. This is consistent with our data that implicated only S546 in having a strong influence on KLP-7 function *in vivo*. In addition, other kinases have been shown to phosphorylate MCAK and regulate its localization and/or activity in mammalian cells, including Cdk1 (Cyclin-dependent kinase 1), PLK1 (Polo-like kinase 1), and PAK1 (p21 activated kinase) [52–54]. Worm homologues exist for all three kinases and they might also contribute to the regulation of KLP-7 *in vivo*.

### Regulation of KLP-7 activity by Aurora kinases

The kinase assays demonstrated that KLP-7 is a direct substrate of AIR-1 and AIR-2 kinases *in vitro*. AIR-1 on its own exhibited weak kinase activity on KLP-7, however, the addition of TPXL-1 resulted in efficient phosphorylation of KLP-7 by AIR-1. Our results showing that TPXL-1 stimulated the phosphorylation of KLP-7 by Aurora kinase *in vitro*, together with the fact that KLP-7, AIR-1 and TPXL-1 all locate at the centrosomes [48], suggest that KLP-7 is a physiological substrate of the active AIR-1/TPXL-1 complex.

Previous work established that Aurora kinases phosphorylate MCAK to regulate its activity with spatial and temporal precision. In the chromatin region, Aurora B kinase phosphorylates MCAK at S196 to inhibit its MT depolymerization activity, and at T95 and S110 to regulate its location to chromatin [8, 10, 16, 17]. In *Xenopus* egg extracts without chromatin and centrosomes, Aurora A kinase phosphorylates MCAK at S196 to promote pole focusing and at S719 to promote its localization to spindle poles for bipolar spindle formation [23]. In this study, we found that individual Aurora kinase sites and groups of up to 8 sites (*e*.*g*., T119E, T119A, S538ES539E, S538AS539A, N4E, N4A, 8A, and T182ES539ES546E) could be altered without affecting the localization of KLP-7 in *C*. *elegans* embryos. Although GFP::KLP-7 fluorescence is specifically reduced at the centrosomes in *air-1(RNAi)* mitotic embryos (S6 Fig), *air-1(RNAi)* embryos are defective in overall centrosome maturation [44], making it difficult to make conclusions about the role of phosphorylation on KLP-7 localization when the kinase is depleted. The GFP::KLP-7 protein that contained 10 mutated Aurora sites (10E) failed to localize to kinetochores and centrosomes. Although we could not rule out the possibility of incorrect folding of the 10E protein *in vivo*, it is possible that dephosphorylation of KLP-7 at multiple Aurora sites is required for proper localization in mitosis.

### A role for S546 in KLP-7 function

Despite localizing normally, GFP::KLP-7(S546A) and GFP::KLP-7(S546E) transgenes failed to rescue *klp-7(tm2143)* embryonic lethality or spindle breakage. The predicted Aurora kinase site lies within the catalytic core and is well conserved among *C*. *elegans*, *Xenopus*, mouse and human (Fig 6D). S546 is located in the α-helix 5 of the catalytic core domain, which is likely important for kinesin-MT binding [55, 56]. Phosphorylation at S546 may introduce a negative charge to this region and thus interfere with KLP-7’s interaction with MTs. Direct evidence for phosphorylation of this particular residue by Aurora is still needed; however, the phospho-mutations reveal an important function for this site.

It is well established that phosphorylation regulates the precise location and function of kinesin-13 family proteins, however, few reports have identified functions for phosphorylation sites in the core domain [8, 10, 16, 17, 23, 57]. The *Drosophila* kinesin-13 protein, KLP10A is phosphorylated by casein kinase 1α in the core domain (*Drosophila* S573, *C*. *elegans* S555). Phosphorylation at this site alters KLP10A association with the MT lattice [57]. One Cdk1 phosphorylation site (T537) has also been identified in the vicinity of S542 (the human equivalent of the worm S546 site) in human cells [52]. Phosphorylation of this site by Cdk1 is required for proper mitotic spindle formation, likely by attenuating MCAK MT-depolymerizing activity and promoting the release of MCAK from the spindle poles [52]. Although *C*. *elegans* KLP-7 does not have an equivalent Cdk1 site, S546 could perform a similar function.

### A model for KLP-7 regulation at the centrosome

The FRAP results revealed a distinct difference between GFP::KLP-7(S546A) and GFP::KLP-7(S546E) mutants. When MTs were depolymerized with nocodazole, GFP::KLP-7(S546A) and GFP::KLP-7(WT) recovery was 2-fold slower compared to untreated embryos, whereas the GFP::KLP-7(S546E) mutant embryos showed no change upon treatment. This suggests that turnover of KLP-7(S546A) at the centrosome is at least somewhat dependent on MTs. Furthermore, in the nocodazole-treated cells, a significant portion of centrosomal KLP-7 is likely not phosphorylated on S546, because the WT protein exhibited recovery rates that were similar to the S546A mutant. However, it is difficult to explain why the S546A mutant did not rescue the MT defects associated with *klp-7(tm2143)*. In order to reconcile this result with the FRAP data, we suggest that KLP-7 undergoes a 2-step process for normal activity (Fig 7D). In this model, S546E and S546A can both locate to the centrosome, but neither form is able to depolymerize centrosomal MTs directly. We suggest that an initial phosphorylation step is required at the centrosome, likely by AIR-1 kinase/TPXL-1 (step 1 in Fig 7D). Then, subsequent dephosphorylation at S546 (step 2 in Fig 7D) would promote MT depolymerization. In this manner, cycles of phosphorylation and dephosphorylation would be required for the normal activity of the protein *in vivo*, explaining why both S546A and S546E exhibit loss-of-function phenotypes. An initial phosphorylation step could allow precise temporal or spatial regulation of a centrosome-specific pool of KLP-7, in order to prevent inappropriate activation of the protein by Aurora kinases at, for instance, other regions of the cell. Further work is required to elucidate the precise mechanism by which S546 alters KLP-7 function at the centrosome.

## Material and Methods

### Nematode culture and strains

*C*. *elegans* were cultured under standard conditions [58]. Temperature-sensitive (ts) strains were upshifted at the L4 stage 24 hours before embryos were collected. Strains used included: Bristol strain N2 (wild-type); EU707 *air-2(or207ts) unc-13(e51)* I; MAS7 *klp-7(tm2143)* III; MAS10 *klp-7(tm2143) unc-119(ky571)* III; MAS91 GFP::β-tubulin; mCherry::histone; MAS112 *klp-7(tm2143)* III; GFP::β-tubulin; mCherry::histone; TH32 γ-tubulin::GFP; GFP::histone. *unc-119 (ky571)* was a gift from Dr. David Pilgrim (University of Alberta, Canada). The original *klp-7(tm2143)* mutant was a gift from Dr. S. Mitani, National Bioresource Project, Japan, and was outcrossed 5 times prior to its use in this study. EU707 and TH32 were obtained from the Caenorhabditis Genetics Center (CGC, funded by NIH Office of Research Infrastructure Programs P40 OD010440). The strains expressing GFP::KLP-7-phospho-mutant transgenes were constructed by high-pressure particle bombardment [59] of MAS10 worms with a plasmid containing GFP tagged *klp*-7 genomic sequence (K11D9.1b), *pie-1* promoter and *unc-119(+)* as a selection marker. The strains generated are listed in S1 Table.

### RNA-Mediated Interference (RNAi)

The feeding clones used for *air-1* and *gpr-1/2* were obtained from the RNAi library [60]. The *tpxl-1* feeding clone was constructed by J. Tegha Dunghu. RNAi was performed using a previously described feeding method [61, 62]. Briefly, L4 larvae were placed on plates seeded with dsRNA producing bacteria for 22–26 hrs at 25°C before being examined. RNAi against *air-1*, *air-2*, and *air-1+air2* was performed by injecting dsRNA into the syncytial gonad of L4 worms [61]. dsRNA was injected at 0.5 mg/mL for each gene.

### Protein expression and *in vitro* kinase assays

The GST-AIR-1, GST-AIR-1(KD), and polycistronic GST-ICP-1 + GST-AIR-2, and GST-ICP-1 + GST-AIR-2(KD) constructs were generously provided by Dr. Jill Schumacher (The University of Texas MD Anderson Cancer Center, Houston, TX). cDNAs of KLP-7-N (K11D9.1b, a.a. 2–189), KLP-7-CC (a.a. 487–689), CC2 (a.a.501-620) and cDNA of TPXL-1(a.a.1-63) were cloned into pDEST-15 vector (Invitrogen) for *in vitro* expression. Mutations at putative sites were introduced by using a QuikChange Site-Directed Mutagenesis Kit (Stratagene).

For each kinase assay reaction, kinase and substrate (5–10 μg each) were incubated in kinase buffer (20 mM HEPES pH7.4, 25 mM beta-glycerophosphate, 10 mM MgCl_2_, 5 mM EGTA, 2 mM DTT, and 0.01 mM ATP) including 5 μCi of γ[32P]-ATP for 30 min at 30°C. Samples were resolved by SDS-PAGE, transferred to nitrocellulose membranes (Pierce, Thermo Scientific) and analyzed by autoradiography. Ponceau-S staining was used to confirm that similar amounts of each substrate were added to the reaction (S3 Fig).

### Phos-tag SDS-PAGE

Phos-tag SDS-PAGE was performed as previously described [63]. Proteins were separated by an 8% polyacrylamide gel containing 25 μM Phos-tag acrylamide (NARD Institute) and 25 μM MnCl_2_, and were transferred to nitrocellulose membrane (Pierce, Thermo Scientific). The resulting membrane was blocked with 10% non-fat milk in TBST, and probed with a rabbit anti-KLP-7 polyclonal antibody (1 μg/mL) [27]. The membrane was washed and incubated with a horseradish peroxidise-conjugated goat anti-rabbit antibody (Bio-Rad Laboratories). ECL western blotting substrate (Pierce, Thermo Scientific) was prepared according to the manufacturer’s instructions and applied to immunoblots. The membrane was exposed to Kodak BioMax XAR film for detection.

Lambda Phosphatase treatment was performed according to the manufacturer’s instructions (New England BioLabs). Worm proteins were extracted in lysis buffer (50 mM HEPES pH 7.4, 1 mM EGTA, 1 mM MgCl_2_, 100 mM KCl, 0.05% NP40, 10% glycerol) supplemented with protease inhibitor cocktail (Mini-Complete, Roche Applied Science). To dephosphorylate proteins, 20 μg of whole worm lysate was incubated with 400 U of phosphatase (10 μL total reaction) for 60 min at 30°C. Reaction was stopped by adding 10 μL of 2X SDS-sample buffer.

### 2-D gel electrophoresis

Immobiline DryStrip (7 cm; pH 7–11 NL for Fig 4 and pH 6–11 for S2 Fig) gels, IPG buffer (pH 7–11 NL, and pH 6–11) and DeStreak Rehydration Solution was used for 2D gel experiments (GE Healthcare). Lysates from N2, *air-1(RNAi)* and *air-2(or207ts)* worms were extracted by sonication in 2D sample buffer (8 M Urea, 4% w/v CHAPS, 2% v/v IPG buffer, 40 mM DTT) supplemented with protease inhibitor cocktail (Mini-Complete, Roche Applied Science). The protein concentration was determined by performing the Bradford assay with protein assay dye reagent (Bio-Rad Laboratories). 120 μg of total protein was loaded to each gel strip by rehydration. Isoelectric focusing (IEF) was performed according to the manufacturer’s instructions. Protein samples were further separated on 10% polyacrylamide gels and transferred to nitrocellulose membranes. The resulting immunoblots were probed with affinity-purified anti-KLP-7 antibody. To dephosphorylate proteins for 2D gel analysis, 100 μg of whole-worm lysate was treated with 2000 U of lambda phosphatase and then prepared in 2D sample buffer.

### Immunofluorescence of stained embryos and live-cell imaging

Confocal microscopy was performed with an X81 motorized inverted microscope (Olympus) equipped with a Yokogawa CSU-10 spinning disk system. Images were collected with a 60× objective (NA 1.42) and a Hamamatsu Orca R2 camera controlled by MetaMorph software (Molecular Devices).

To perform immunochemical staining, embryos were processed by freeze-cracking and fixed with methanol as described [27]. Mouse monoclonal anti-α-tubulin antibody, DM1A (Sigma-Aldrich) and rabbit polyclonal anti-KLP-7 antibodies [27] were used at 1 μg/mL and 20 μg/mL respectively. Directly-labelled anti-TAC-1-Cy5 antibodies were used for S1 Fig, as described [64]. Z-stack images were acquired at a step size of 0.2 μm for each meiotic embryo.

To quantify centrosomal MTs in metaphase embryos, GFP::KLP-7(S546E) or GFP::KLP-7(S546A) worms were fixed and stained under the same conditions with the wild-type control worms. DM1A (Sigma-Aldrich) and rabbit polyclonal anti-γ-tubulin antibodies [44] were used at 1 μg/mL and 8 μg/mL respectively. In a single frame where the centrosome is in focus, the centrosomal MT level is defined as the fluorescence intensity of a circular region at the centrosome subtracting the intensity at a background cytoplasmic area. The relative intensity for each centrosome was calculated as: (integrated intensity of one centrosome/the average integrated intensity of control centrosomes) x 100.

GFP::tubulin fluorescence at the midzone of anaphase embryos was quantified from integrated fluorescence intensities within a rectangular region (4.65 x 3.72 μm) from a single confocal plane.

The spindle-snap phenotype was scored by first dissecting worms in 5 μL egg buffer (188 mM NaCl, 48 mM KCl, 2 mM CaCl_2_, 2 mM MgCl_2_, and 25 mM HEPES, pH 7.3). Embryos were then mounted on a thin 2% agarose pad and imaged for GFP::KLP-7(WT or mutants) from the start of mitotic metaphase with 5 second intervals. The environment temperature during imaging was controlled at 25 ± 0.5°C according to previous description [65]. The maximum velocity of the posterior centrosome during the first 40 seconds of mitotic anaphase was determined.

To perform *in utero* imaging of meiotic embryos, worms expressing GFP::tubulin and mCherry-histone were immobilized with 4 mM tetramisole hydrochloride (Sigma-Aldrich) and mounted on a thin 2% agarose pad. 20 second time-lapse images were collected with a 60× silicone oil objective (NA 1.3) from fertilization to the end of meiosis II. At each time point, 3 images were taken at an interval of 1 μm to visualize the structure of the meiotic spindles.

### Nocodazole treatment and FRAP

To create permeabilized embryos, L4-adult worms were grown for 12–16 h at 20°C on *ptr-1* RNAi-feeding plates [66, 67]. Worms were dissected in embryo buffer and mounted on agarose pads with a coverslip for imaging. To depolymerize MTs, nocodazole was added (10 μM final concentration) just before mounting and allowed to incubate for at least 30 seconds [68]. One-cell embryos in metaphase were used for all photobleaching experiments. Only embryos that displayed a lack of anaphase pole separation after the FRAP experiment were deemed affected by nocodazole and used for analysis.

Photobleaching experiments were performed on a spinning disk confocal as described above. The bleaching laser (400 mW; Power Technology) was controlled by Metamorph Software using the Mosaic targeted illumination interface. Sufficient photobleaching of a circle (30 pixel diameter) was achieved with a targeted widefield beam at maximum laser power for 3 seconds. Confocal images were acquired at 1 second intervals both before photobleaching (5 frames), and during the recovery phase (90 frames). Fluorescence intensity of GFP::KLP-7 was measured with Metamorph software at the centrosome and at two background locations to correct for photobleaching resulting from image acquisition. Curves (S8 Fig) were generated with easyFRAP software, using full-scale normalization and double term curve-fitting [69].

## Supporting Information

S1 Fig KLP-7 is not detected in *klp-7(tm2143)* embryos via immunostaining. Embryos were fixed and immunostained with anti-tubulin, anti-KLP-7, and anti-TAC-1 antibodies. DNA was visualized with DAPI.
(TIF)

S2 Fig KLP-7 is phosphorylated *in vivo*. 2D gel electrophoresis of wild-type lysate (top) and wild-type lysate treated with phosphatase (bottom) followed by immunoblotting with anti-KLP-7 antibodies. pH gradient is indicated. Arrow: non-phosphorylated form of KLP-7. White bracket: phospho-isoforms of KLP-7, which are reduced or eliminated by phosphatase treatment. Note: the pH range differs from that depicted in Fig 4.
(TIF)

S3 Fig Loading controls for *in vitro* kinase assays. Ponceau S staining of membranes was used to assess protein loading for the *in vitro* kinase assay results shown in Fig 5. Panels A-D correspond to Fig 5B–5E. The same membrane was preceded with autoradiography to detect phosphorylation of KLP-7.
(TIF)

S4 Fig A large C-terminal fragment of KLP-7, KLP-7-CC, is not effectively phosphorylated by AIR-2/ICP-1 *in vitro*. AIR-2 kinase and co-activator ICP-1 were incubated with different substrates *in vitro* and γ[32P]-ATP incorporation was determined via SDS-PAGE and subsequent autoradiography. Left: AIR-2/ICP-1 phosphorylates GST-KLP-7-N (arrowhead) but not GST-KLP-7-CC (predicted MW of 49 KDa). Middle: The kinase dead AIR-2-KD/ICP-1 does not phosphorylate KLP-7-N nor KLP-7-CC. Right: AIR-2/ICP-1 does not phosphorylate GST alone. AIR-2 auto-phosphorylation is shown by using H_2_O instead of substrate.
(TIF)

S5 Fig Non-rescuing mutant *klp-7* transgenes are expressed. The expression levels of GFP::KLP-7(S546E), GFP::KLP-7(S546A) and GFP::KLP-7(10E) transgenes were determined by western blotting. Transgenic worm lysates were probed with anti-tubulin and anti-KLP-7 antibodies. Seventy young adult hermaphrodites were loaded for each lane.
(TIF)

S6 Fig KLP-7 localization after Aurora kinase knock-down. Wild-type and *air-1(RNAi)* mitotic embryos were imaged for GFP::KLP-7 fluorescence. The *air-1(RNAi)* embryos were isolated from worms subjected to RNA-feeding for 14 or 24 hours. Selected time-points are shown; time is relative to the initiation of a mitotic cytokinesis furrow. GFP-KLP-7 levels are reduced in *air-1(RNAi)* embryos specifically at the centrosomes (arrowheads).
(TIF)

S7 Fig Phospho-mutant *klp-7* transgenes that rescue the spindle-break phenotype of *klp-7(tm2143)*. Different versions of KLP-7-GFP were expressed in *klp-7(tm2143)* worms and the maximum velocity of the posterior centrosome during the first 40 seconds of mitotic anaphase was determined. The relevant alteration to the *klp-7* transgene is listed. The average (based on n centrosomes) is plotted for GFP-KLP-7(WT), P = 1.0; *klp-7(tm2143)*, P = 0.002; GFP-KLP-7(8A), P = 0.26; GFP::KLP-7(N4E), P = 0.24; GFP::KLP-7(N4A), P = 0.09; GFP::KLP-7(T119E), P = 0.51; GFP::KLP-7(T119A), P = 0.18; GFP::KLP-7(S538ES539E), P = 0.16; GFP::KLP-7(S538AS539A), P = 0.83; GFP::KLP-7(S539E), P = 0.28; GFP::KLP-7(S539A), P = 0.97. *klp-7(tm2143)* control worms expressed GFP::γ-tubulin and GFP::histone to enable centrosome tracking; all other strains were tracked with the GFP::KLP-7 signal. Error bars are SEM. P-values were based on two-tailed Student’s t-tests comparing each mutant to wild type. ** (0.0005<P<0.005).
(TIF)

S8 Fig FRAP Curves. Fitted curves (red lines) are shown with the averaged data (blue dots) for each experimental condition. Relevant KLP-7 mutations are indicated.
(TIF)

S1 File Raw data of the results presented in Fig 1C. (XLSX)

S2 File Raw data of the results presented in Fig 2B. (XLSX)

S3 File Raw data of the results presented in Fig 3B. (XLSX)

S4 File Raw data of the results presented in Fig 6B. (XLSX)

S5 File Raw data of the results presented in Fig 6C. (XLSX)

S6 File Raw data of the results presented in Fig 7B and 7C. (XLSX)

S7 File Raw data of the results presented in S7 Fig. (XLSX)

S8 File Raw data of the results presented in Table 1. (XLSX)

S9 File Raw data of the results presented in Table 2. (XLSX)

S1 Movie A time-lapse movie showing female meiotic divisions (*in utero*) of a wild-type embryo expressing GFP::tubulin and mCherry::histone transgenes. Time (min:sec) is relative to start of MI anaphase.
(MOV)

S2 Movie A time-lapse movie showing female meiotic divisions (*in utero*) of a *klp-7(tm2143)* embryo expressing GFP::tubulin and mCherry::histone transgenes. Time (min:sec) is relative to start of MI anaphase.
(MOV)

S3 Movie The first mitosis of a wild-type embryo expressing GFP::γ-tubulin and GFP::histone transgenes. (MOV)

S4 Movie *klp-7(tm2143)* embryo expressing GFP::γ-tubulin and mCherry::histone transgenes exhibits a spindle-break phenotype. (MOV)

S5 Movie GFP::KLP-7(WT) transgene rescues the spindle-break phenotype of *klp-7(tm2143)*. (MOV)

S1 Table Genotype of phospho-mutant KLP-7 transgenic lines. (DOCX)
