# Supplementary figures and images for: The KLP-7 Residue S546 Is a Putative Aurora Kinase Site Required for Microtubule Regulation at the Centrosome in C. elegans
Source: PLoS One. 2015 Jul 13;10(7):e0132593. doi: 10.1371/journal.pone.0132593 (PMC4500558; doi:10.1371/journal.pone.0132593)

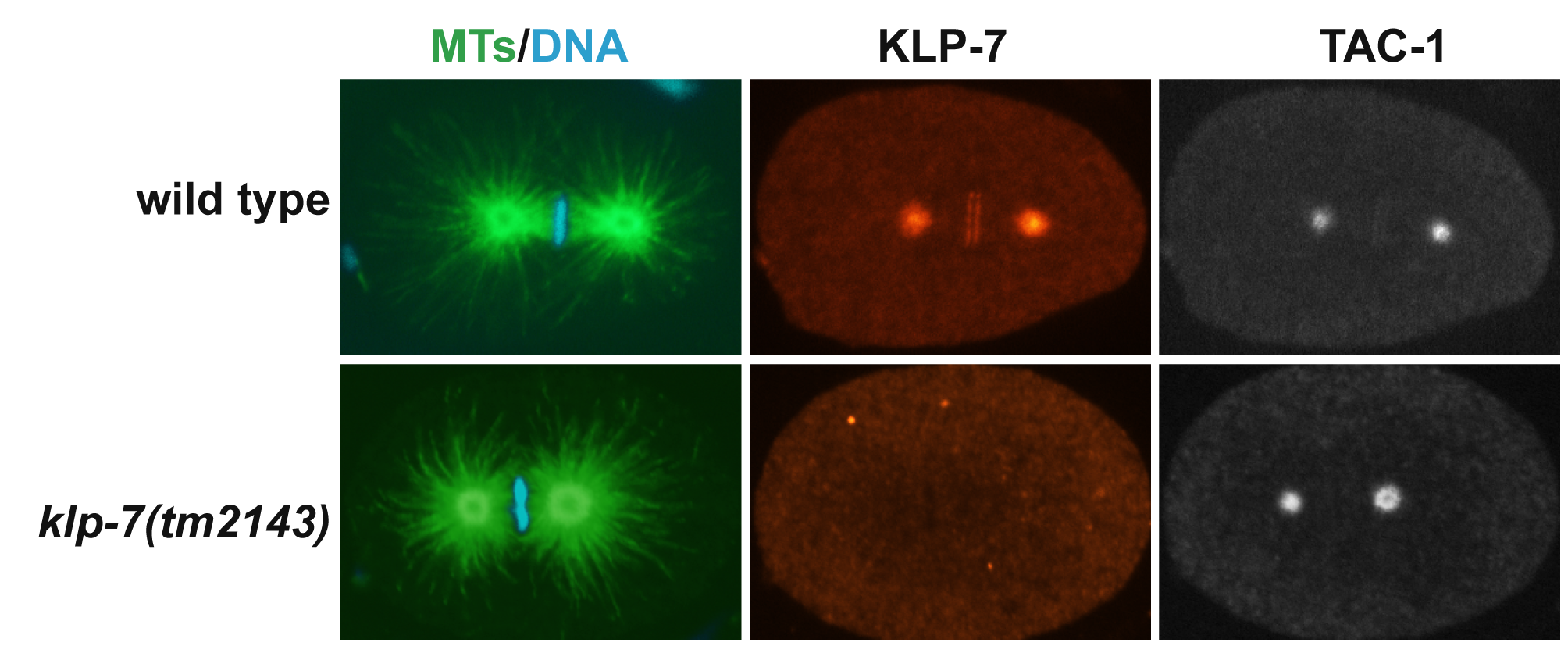

Supplement: S1 Fig — Embryos were fixed and immunostained with anti-tubulin, anti-KLP-7, and anti-TAC-1 antibodies. DNA was visualized with DAPI. (TIF) [file pone.0132593.s001.tif]

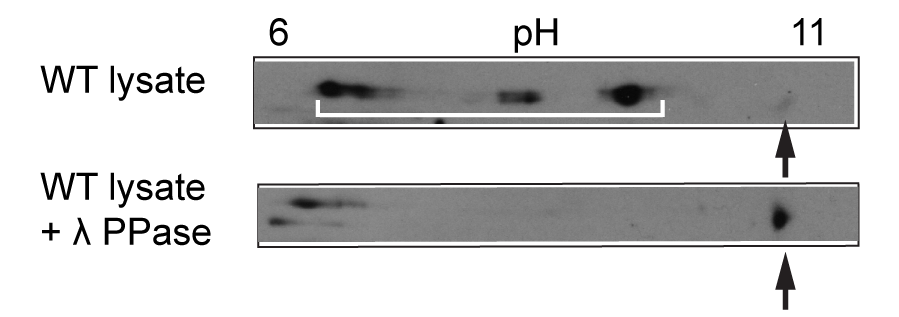

Supplement: S2 Fig — 2D gel electrophoresis of wild-type lysate (top) and wild-type lysate treated with phosphatase (bottom) followed by immunoblotting with anti-KLP-7 antibodies. pH gradient is indicated. Arrow: non-phosphorylated form of KLP-7. White bracket: phospho-isoforms of KLP-7, which are reduced or eliminated by phosphatase treatment. Note: the pH range differs from that depicted in Fig 4. (TIF) [file pone.0132593.s002.tif]

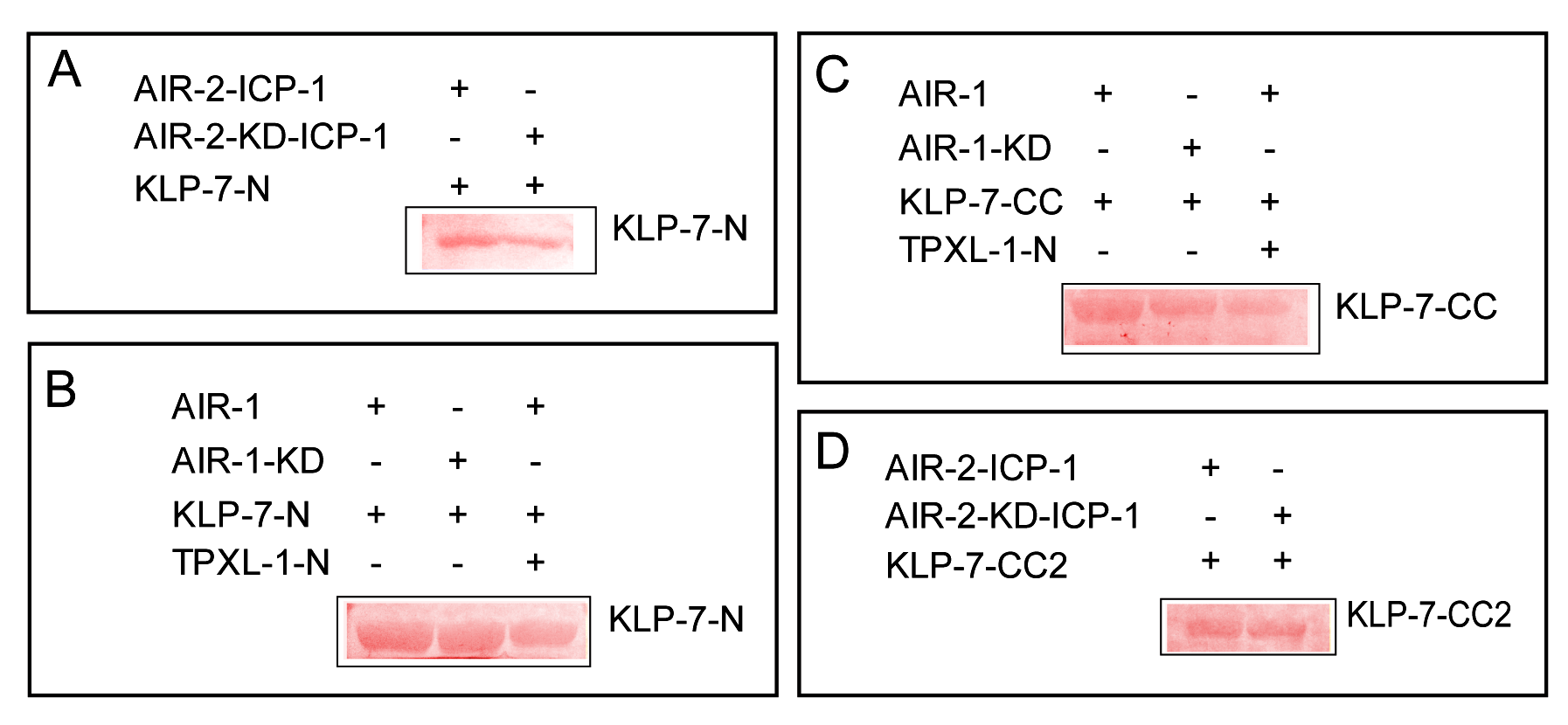

Supplement: S3 Fig — Ponceau S staining of membranes was used to assess protein loading for the in vitro kinase assay results shown in Fig 5. Panels A-D correspond to Fig 5B–5E. The same membrane was preceded with autoradiography to detect phosphorylation of KLP-7. (TIF) [file pone.0132593.s003.tif]

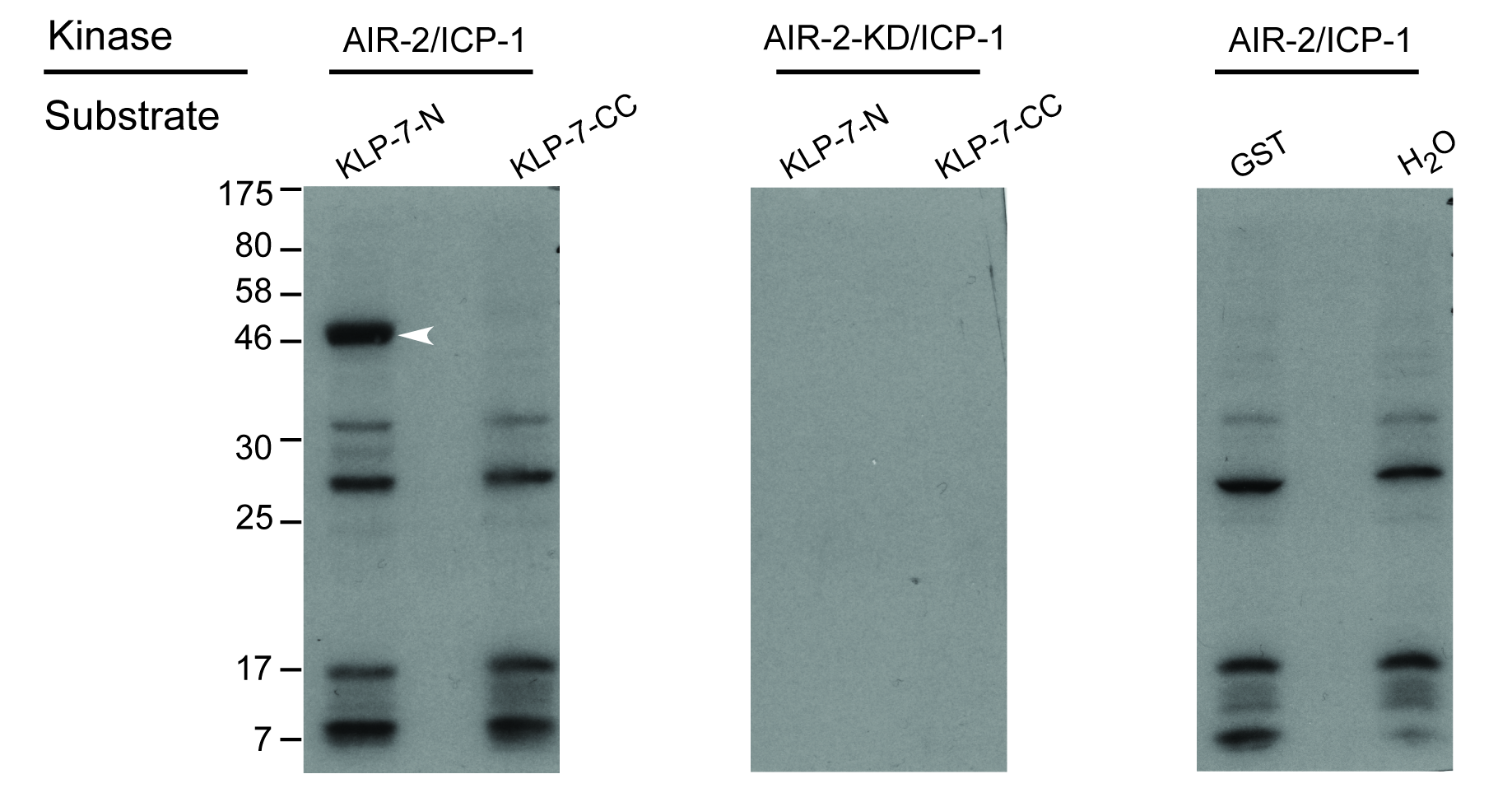

Supplement: S4 Fig — AIR-2 kinase and co-activator ICP-1 were incubated with different substrates in vitro and γ[32P]-ATP incorporation was determined via SDS-PAGE and subsequent autoradiography. Left: AIR-2/ICP-1 phosphorylates GST-KLP-7-N (arrowhead) but not GST-KLP-7-CC (predicted MW of 49 KDa). Middle: The kinase dead AIR-2-KD/ICP-1 does not phosphorylate KLP-7-N nor KLP-7-CC. Right: AIR-2/ICP-1 does not phosphorylate GST alone. AIR-2 auto-phosphorylation is shown by using H2O instead of substrate. (TIF) [file pone.0132593.s004.tif]

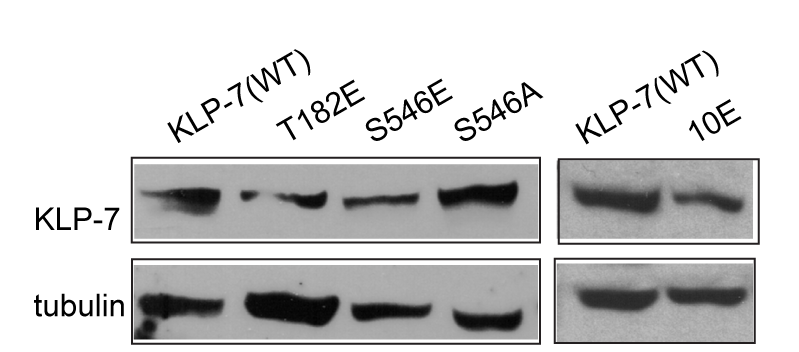

Supplement: S5 Fig — The expression levels of GFP::KLP-7(S546E), GFP::KLP-7(S546A) and GFP::KLP-7(10E) transgenes were determined by western blotting. Transgenic worm lysates were probed with anti-tubulin and anti-KLP-7 antibodies. Seventy young adult hermaphrodites were loaded for each lane. (TIF) [file pone.0132593.s005.tif]

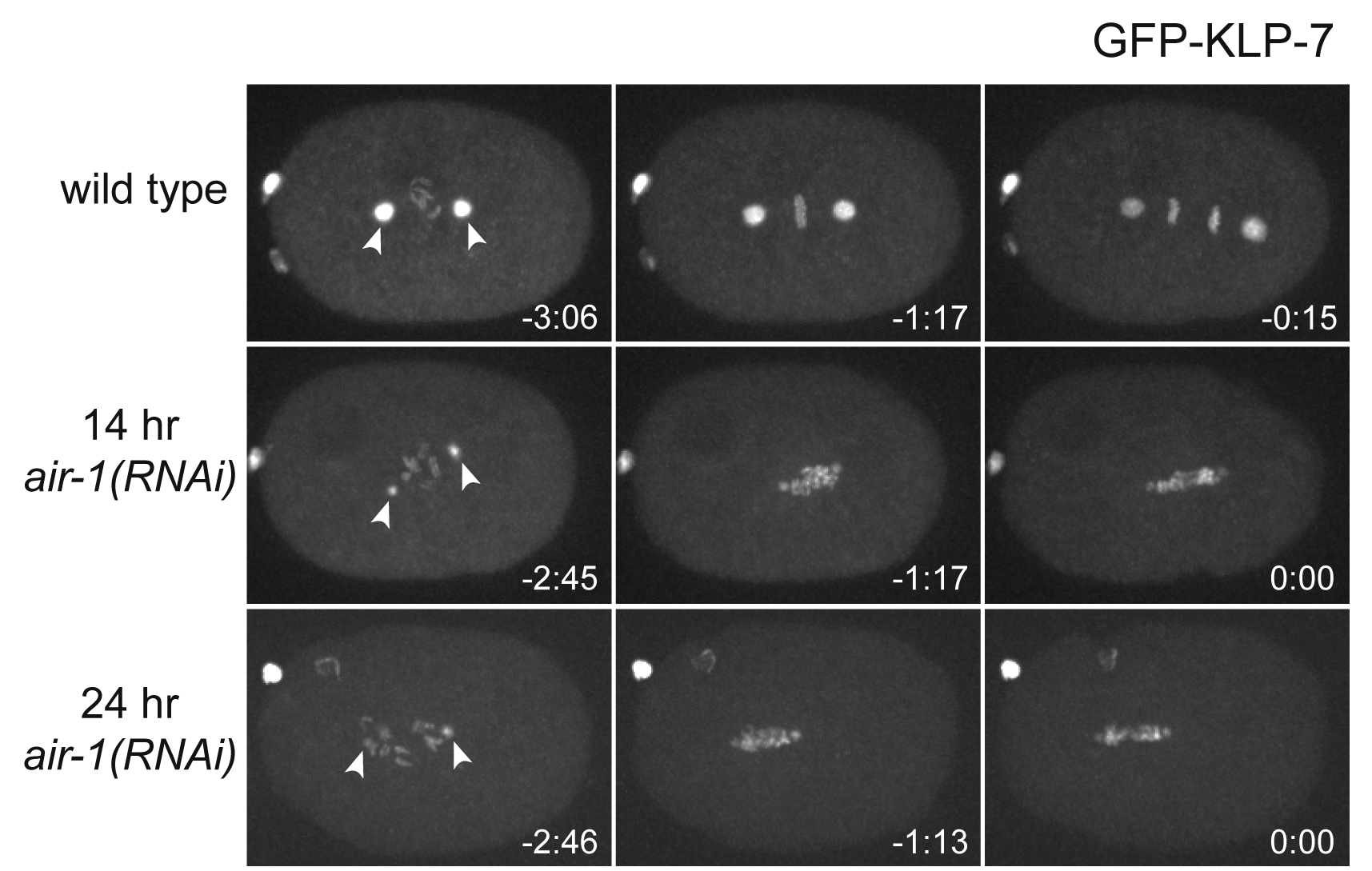

Supplement: S6 Fig — Wild-type and air-1(RNAi) mitotic embryos were imaged for GFP::KLP-7 fluorescence. The air-1(RNAi) embryos were isolated from worms subjected to RNA-feeding for 14 or 24 hours. Selected time-points are shown; time is relative to the initiation of a mitotic cytokinesis furrow. GFP-KLP-7 levels are reduced in air-1(RNAi) embryos specifically at the centrosomes (arrowheads). (TIF) [file pone.0132593.s006.tif]

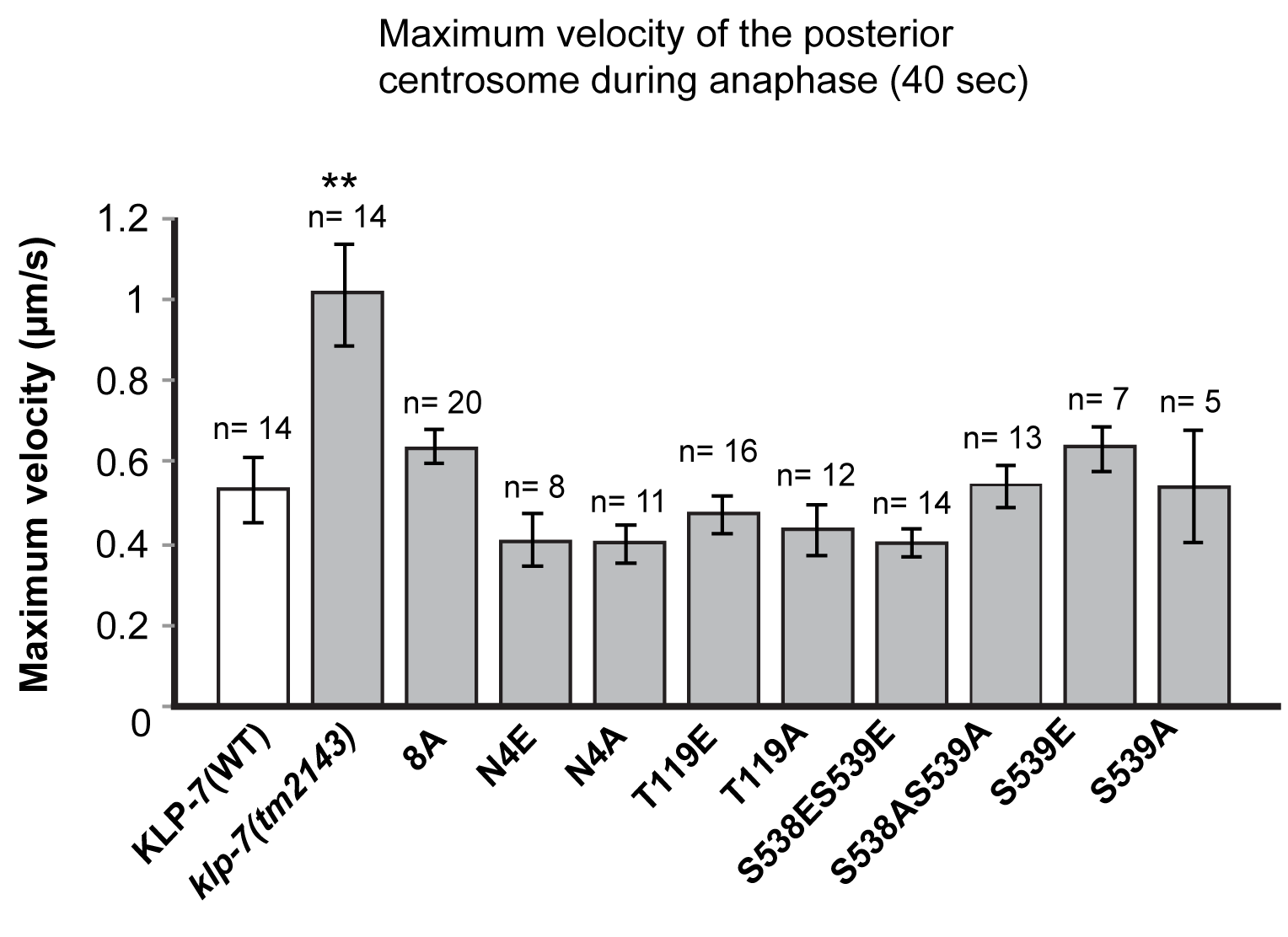

Supplement: S7 Fig — Different versions of KLP-7-GFP were expressed in klp-7(tm2143) worms and the maximum velocity of the posterior centrosome during the first 40 seconds of mitotic anaphase was determined. The relevant alteration to the klp-7 transgene is listed. The average (based on n centrosomes) is plotted for GFP-KLP-7(WT), P = 1.0; klp-7(tm2143), P = 0.002; GFP-KLP-7(8A), P = 0.26; GFP::KLP-7(N4E), P = 0.24; GFP::KLP-7(N4A), P = 0.09; GFP::KLP-7(T119E), P = 0.51; GFP::KLP-7(T119A), P = 0.18; GFP::KLP-7(S538ES539E), P = 0.16; GFP::KLP-7(S538AS539A), P = 0.83; GFP::KLP-7(S539E), P = 0.28; GFP::KLP-7(S539A), P = 0.97. klp-7(tm2143) control worms expressed GFP::γ-tubulin and GFP::histone to enable centrosome tracking; all other strains were tracked with the GFP::KLP-7 signal. Error bars are SEM. P-values were based on two-tailed Student’s t-tests comparing each mutant to wild type. ** (0.0005<P<0.005). (TIF) [file pone.0132593.s007.tif]

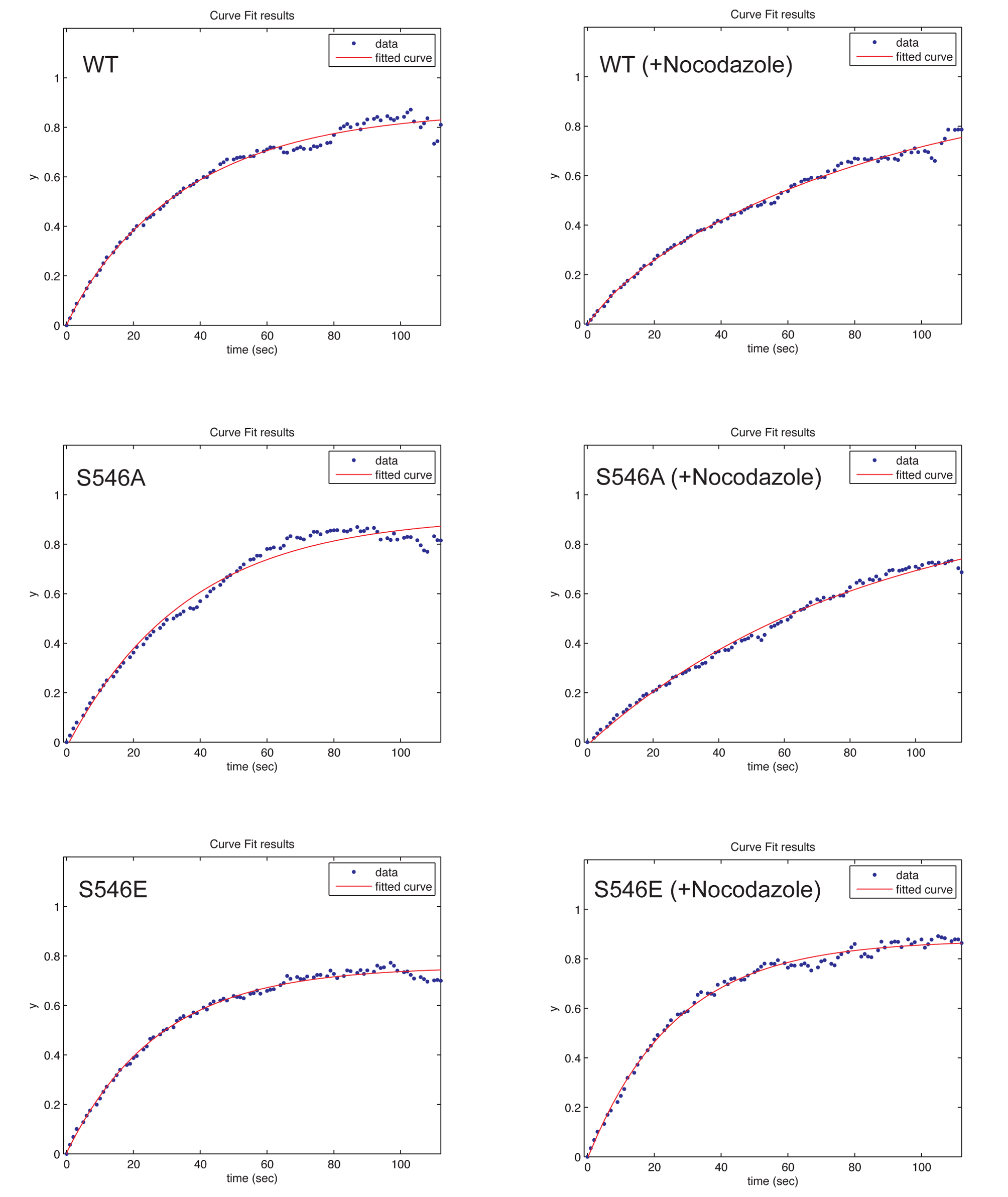

Supplement: S8 Fig — Fitted curves (red lines) are shown with the averaged data (blue dots) for each experimental condition. Relevant KLP-7 mutations are indicated. (TIF) [file pone.0132593.s008.tif]
