# Supplementary material for: The KLP-7 Residue S546 Is a Putative Aurora Kinase Site Required for Microtubule Regulation at the Centrosome in C. elegans
Source: PLoS One. 2015 Jul 13;10(7):e0132593. doi: 10.1371/journal.pone.0132593 (PMC4500558; doi:10.1371/journal.pone.0132593)
Supplement: S1 Table — (DOCX) [file pone.0132593.s023.docx]

**Table S1: genotype of phospho-mutant KLP-7 transgenic lines**

| Name in text and figures | Genotype |
| --- | --- |
| KLP-7 WT | MAS42, *unc-119(ky571) klp-7(tm2143); Is20 [pie-1::GFP::KLP-7(WT); unc-119(+)]* |
| T119E | MAS152, *unc-119(ky571) klp-7(tm2143); Is42 [pie-1::GFP::KLP-7(T119E); unc-119(+)]* |
| T119A | MAS149, *unc-119(ky571) klp-7(tm2143); Is39 [pie-1::GFP::KLP-7(T119A); unc-119(+)]* |
| N4E | MAS156, *unc-119(ky571) klp-7(tm2143); Is44 [pie-1::GFP::KLP-7(T119ET159ET160ET182E); unc-119(+)]* |
| N4A | MAS145, *unc-119(ky571) klp-7(tm2143); Is36 [pie-1::GFP::KLP-7(T119AT159AT160AT182A); unc-119(+)]* |
| S539E | MAS29, *unc-119(ky571) klp-7(tm2143); Is13 [pie-1::GFP::KLP-7(S539E); unc-119(+)]* |
| S539A | MAS86, *unc-119(ky571) klp-7(tm2143); Is27 [pie-1::GFP::KLP-7(S539A); unc-119(+)]* |
| S538ES539E | MAS148, *unc-119(ky571) klp-7(tm2143); Is38 [pie-1::GFP::KLP-7(S538ES539E); unc-119(+)]* |
| S538AS539A | MAS143, *unc-119(ky571) klp-7(tm2143); Is35 [pie-1::GFP::KLP-7(S538AS539A); unc-119(+)]* |
| S546E | MAS31, *unc-119(ky571) klp-7(tm2143); Is15 [pie-1::GFP::KLP-7(S546E); unc-119(+)]* |
| S546A | MAS92, *unc-119(ky571) klp-7(tm2143); Is28 [pie-1::GFP::KLP-7(S546A); unc-119(+)]* |
| T182ES539ES546E | MAS52, *unc-119(ky571) klp-7(tm2143); Is24 [pie-1::GFP::KLP-7(T182ES539ES546E); unc-119(+)]* |
| T182E | MAS25, *unc-119(ky571) klp-7(tm2143); Is9 [pie-1::GFP::KLP-7(T182E); unc-119(+)]* |
| 8A | MAS174, *unc-119(ky571) klp-7(tm2143); Is51 [pie-1::GFP::KLP-7(T119AT159AT160AT182AS538AS539AT652AT653A); unc-119(+)]* |
| 10E | MAS168, *unc-119(ky571) klp-7(tm2143); Is48 [pie-1::GFP::KLP-7(T119ET159ET160ET182ES538ES539ES546ES555ET652ET653E); unc-119(+)]* |
